# To 200,000 *m*/*z* and Beyond: Native Electron Capture Charge Reduction Mass Spectrometry Deconvolves Heterogeneous Signals in Large Biopharmaceutical Analytes

**DOI:** 10.1021/acscentsci.4c00462

**Published:** 2024-07-26

**Authors:** Kyle I. P. Le Huray, Tobias P. Wörner, Tiago Moreira, Marcin Dembek, Maria Reinhardt-Szyba, Paul W. A. Devine, Nicholas J. Bond, Kyle L. Fort, Alexander A. Makarov, Frank Sobott

**Affiliations:** † Astbury Centre for Structural Molecular Biology, School of Molecular and Cellular Biology, Faculty of Biological Sciences, University of Leeds, Woodhouse Lane, Leeds LS2 9JT, U.K.; ‡ Thermo Fisher Scientific (Bremen) GmbH, Hanna-Kunath Str. 11, 28199 Bremen, Germany; § Purification Process Sciences, Biopharmaceutical Development, Biopharmaceuticals R&D, AstraZeneca, 1 Francis Crick Avenue, Cambridge CB2 0AA, U.K.; ∥ Analytical Sciences, Biopharmaceutical Development, Biopharmaceuticals R&D, AstraZeneca, 1 Francis Crick Avenue, Cambridge CB2 0AA, U.K.; ⊥ Biomolecular Mass Spectrometry and Proteomics, Bijvoet Centre for Biomolecular Research and Utrecht Institute for Pharmaceutical Sciences, Utrecht University, Padualaan 8, 3584 CH Utrecht, The Netherlands

## Abstract

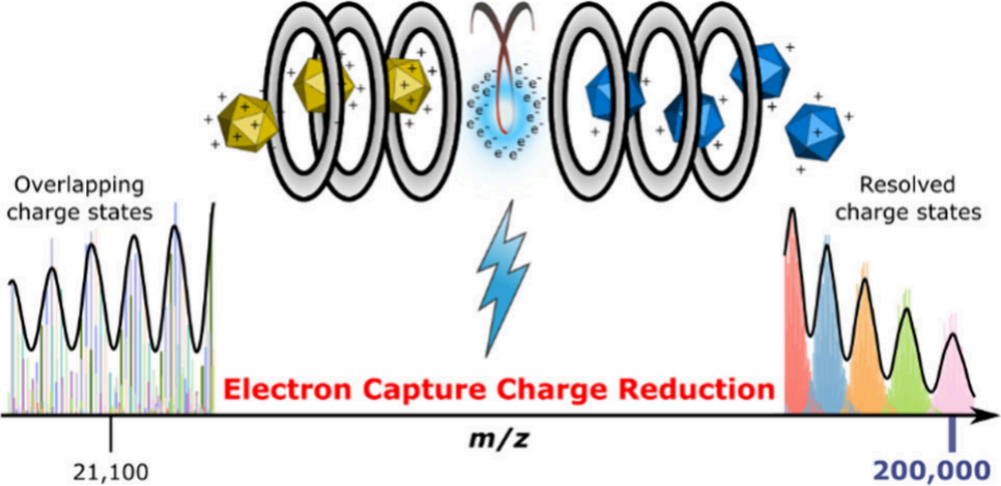

Great progress has been made in the detection of large biomolecular analytes by native mass spectrometry; however, characterizing highly heterogeneous samples remains challenging due to the presence of many overlapping signals from complex ion distributions. Electron-capture charge reduction (ECCR), in which a protein cation captures free electrons without apparent dissociation, can separate overlapping signals by shifting the ions to lower charge states. The concomitant shift to higher *m*/*z* also facilitates the exploration of instrument upper *m*/*z* limits if large complexes are used. Here we perform native ECCR on the bacterial chaperonin GroEL and megadalton scale adeno-associated virus (AAV) capsid assemblies on a Q Exactive UHMR mass spectrometer. Charge reduction of AAV8 capsids by up to 90% pushes signals well above 100,000 *m*/*z* and enables charge state resolution and mean mass determination of these highly heterogeneous samples, even for capsids loaded with genetic cargo. With minor instrument modifications, the UHMR instrument can detect charge-reduced ion signals beyond 200,000 *m*/*z*. This work demonstrates the utility of ECCR for deconvolving heterogeneous signals in native mass spectrometry and presents the highest *m*/*z* signals ever recorded on an Orbitrap instrument, opening up the use of Orbitrap native mass spectrometry for heavier analytes than ever before.

## Abstract 2

Mass spectra of large heterogeneous protein complexes can exhibit unassignable signal overlap. Extreme electron capture charge reduction can separate these signals by shifting the ions to higher *m*/*z*.

## Introduction

Native mass spectrometry involves the ionization and solution-to-gas phase transfer of biological macromolecules and complexes such as proteins and nucleic acids, while preserving a near-native structure and maintaining noncovalent interactions.^1−4^ Biological assemblies studied in this way range from small protein–ligand complexes to diverse and heterogeneous ribosomes, intact viruses, DNA nanostructures, and protein–lipid supercomplexes released directly from membranes.^5−9^ The number of charges acquired by a biomolecular analyte during nanoelectrospray ionization (nano-ESI) depends primarily on its solution-phase solvent accessible surface area and the chemical composition of the solution.^10,11^ Manipulation of the charge state distribution, either to reduce (charge reduction) or to increase (supercharging) the number of charges on the analyte, can be desirable in multiple use-cases. In cases where the analyte natively charges by nano-ESI at a mass-to-charge ratio (*m*/*z*) range beyond the capabilities of an instrument, charge manipulation can be used to move the *m*/*z* of the analyte into the detectable range.^12^ Electric fields are used to control ions in the mass spectrometer, and in combination with a background collision gas, ions can be accelerated to cause collisional heating, for example, to achieve fragmentation, dissociation, or desolvation; the extent of acceleration and therefore activation at a given voltage is related to the ion’s charge, and charge manipulation can therefore help to control the extent of activation. Supercharging has for example been shown to assist in desolvation for some membrane proteins, for which greater activation is needed to eject the protein from detergent micelles; however, in other cases, charge reduction is useful to prevent unfolding of the protein due to overactivation or internal Coulomb repulsion, and is also of fundamental interest for understanding protein structure in the gas phase.^11,13−18^

**Figure 1:**
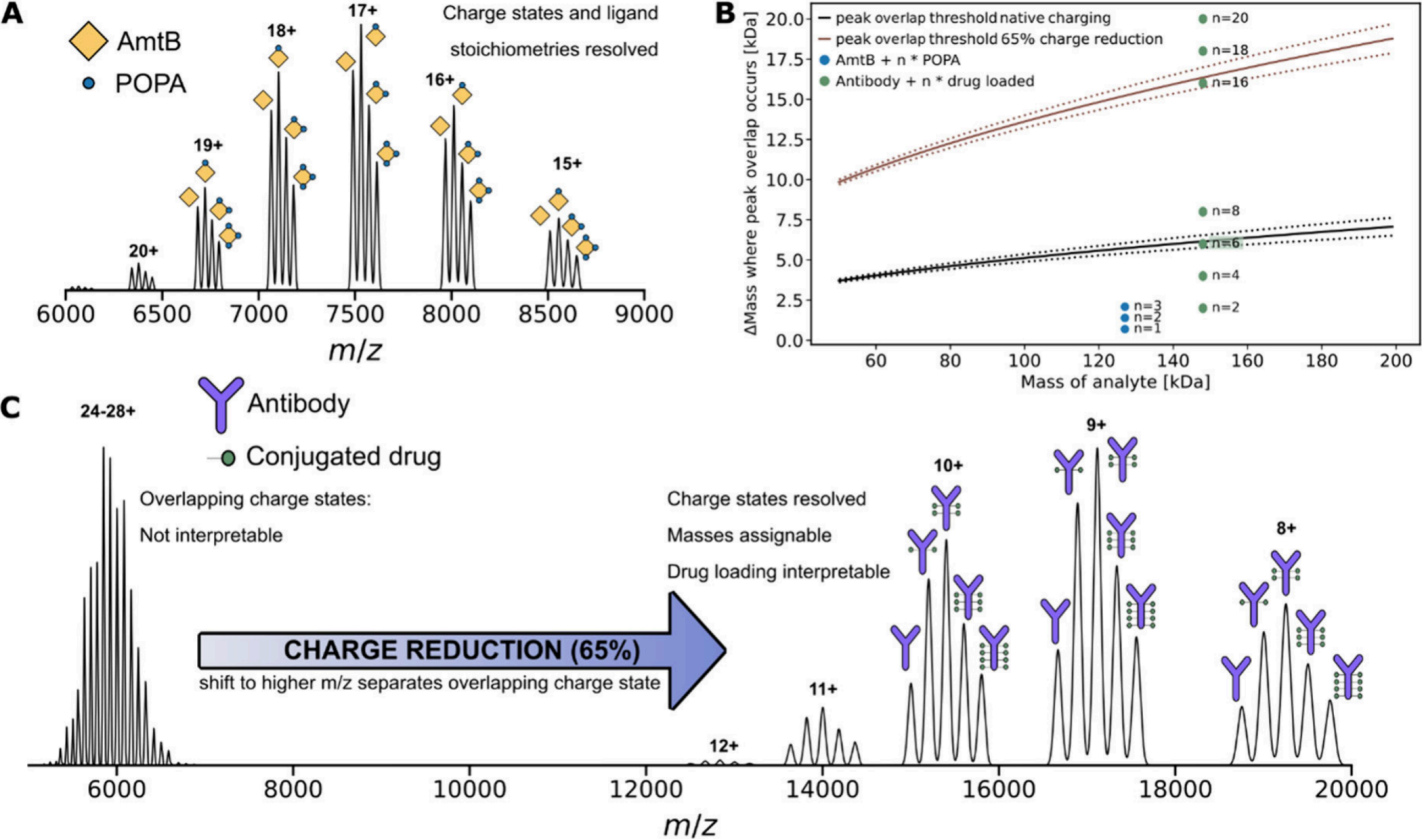
Simulations illustrating how charge reduction can separate overlapping charge state distributions in native mass spectrometry. (A) simulated native mass spectrum of ammonium transporter AmtB, for which charge states and lipid adducts (POPA) can be resolved without charge reduction, as an example of the ideal case in native mass spectrometry. (B) Predicted overlap of adjacent charge state peaks for ligand binding of AmtB (blue circles, *n* = number of bound) and for covalent drug loading of an antibody (green circles, *n* = number of loaded drugs). The *z* and *z* + 1 charge states of two ions of different mass will overlap when the corresponding Δmass value lies in the zone between the dotted lines, as is the case here for the *n* = 6 drug-loaded antibody. Black lines were calculated on the basis of eq 2 (assuming native charging behavior, SI Figure 1). Brown lines are based on moderate (65%) charge reduction. Solid lines indicate the Δmass where exact signal overlap will arise while dotted lines bound a window of unresolvable peak overlap due to instrument limitations (here modeled for an Orbitrap analyzer at resolution setting 1500, as a low resolution example). (C) simulated native mass spectrum of an antibody–drug conjugate showing overlapping charge states (signal around 6000 *m*/*z*) with native charging, precluding interpretation; charge reduction increases the separation between adjacent charge states, enabling charge state resolution and inference of the DAR.^22^ All simulations are modeled on the basis of real spectra reported in the literature.^21−23^

A major use-case for charge reduction in native mass spectrometry is to attain separation of overlapping peaks resulting from the heterogeneity present in many biological macromolecules. The ideal native mass spectrum yields a fully resolved distribution of signals for each species over different charge states; assignment of ion charge from the distribution enables mass calculation and ideally resolves mass heterogeneity resulting from, for example, ligand binding (Figure 1A). As long as neighboring charge states do not overlap with each other and the ions are sufficiently desolvated, a high resolving power of the mass analyzer should enable the resolution of even the smallest mass differences, up to the isotopic fine structure within the analytes.^19^ However, as analytes increase in size and complexity, the mass differences between individual species can become difficult to resolve where the ions of neighboring charge state distributions merge into each other, and ions of different charge states coincide exactly at the same *m*/*z* position or at close enough *m*/*z* position to cause peak overlap unresolvable with the available resolving power of the mass analyzer. This is most often the case in samples where the analyte can harbor several modifications which increase the mass, so that it merges into the adjacent charge state and (eq 1) is fulfilled:
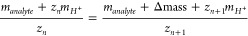
1where *m*_*analyte*_ is the mass of the analyte of charge *z*_*n*_, which will have exact peak overlap with the *z*_*n*+1_ charge state of an ion with additional mass, Δ*mass*. Rearranging this, we can calculate at which Δmass complete peak overlap would occur, which would be unresolvable even at near-infinite resolving power:
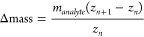
2In the case of neighboring charge states of course *z*_*n*+1_ – *z*_*n*_ = 1, and eq 2 can be simplified as . Assuming the average native charge at a given mass (Supporting Information (SI) Figure 1), we can simulate how Δmass scales with the analyte mass, plotted as the solid line black line in Figure 1B.^7,20^ The resolving power of the mass analyzer furthermore adds a window of overlap to Δmass (see dotted lines in Figure 1B), as for two peaks to be resolved by the instrument the following equation needs to be fulfilled: 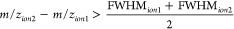
As an example of the ideal case in native mass spectrometry, for ammonia transporter AmtB and its POPA (1-palmitoyl-2-oleoyl-*sn*-glycero-3-phosphate) lipid ligands, it can clearly be seen in Figure 1B that, at its given native charging, all ligand-bound states lie well below this Δmass region where peak overlap occurs, no overlapping charge states are present, and ligand peaks are well resolved in Figure 1A.^21^ However, for an antibody–drug conjugate (ADC) with different numbers of drugs loaded, native charging and the larger size of loaded drugs predict that the ions of the two neighboring drug-to-antibody ratio (DAR) variants 0 and 6 will fall within the peak overlap window and will be inseparable on lower resolution mass analysers, as reported experimentally.^22^ This behavior will result in mixed charge state distributions, which are often elusive for mass determination and quantification, as it is not apparent from the overlapping signals how many ion species are hiding within one peak (Figure 1C and SI Figure 2). Moderate charge reduction resolves this by moving the peak overlap threshold to higher Δmass.

The effect of charge reduction can also be simulated using eq 2, to estimate at which drug loading level (*Δmass*) peak overlap will appear again (Figure 1B). In addition to ADCs, charge reduction has been exploited for separation of overlapping signals and characterization of for example, vaccine components, synthetic polymers, and membrane proteins.^16,22,24−29^ For modeling electron capture charge reduction, eq 2 should strictly be adapted to account for the mass of one additional proton per reduced charge. This is because during positive mode electrospray ionization, the protein is charged by protonation of surface-exposed amino acids. Therefore, a protein ion charge reduced by electron capture to a lower charge state will carry with it additional proton mass compared to an ion of the same protein which had been ionised to the lower state without having undergone charge reduction. However, this additional mass is negligible for the kilodalton and megadalton sized analytes discussed herein.

For the example in Figure 1B, we considered only a single charge state (the assumed average native charge state). Where other charge states are present in the spectrum (which have a different Δmass for peak overlap), it may be possible to make assignments from these other charge states; however, heterogeneity from, for example, solvent adducts or glycoforms (in the case of ADCs) may make this more difficult. Furthermore, as analytes become heavier and/or multimeric, heterogeneity can also arise from the presence of many different types of ligands required for their biological functions, although these can often still be resolved with high instrument resolution or the use of chemical charge reduction.^30^ A further level of complexity can arise in the megadalton scale with analytes such as virus-like particles and nanocages, which can have high numbers of subunits and large encapsulated cargo molecules. Stochastic assembly of different subunits and/or variations in cargo loading can result in extreme heterogeneity in such systems, which can be beyond the capability of existing mass analyzers to resolve. For example, adeno-associated virus (AAV) capsids are particles in the megadalton range used as vectors for gene therapy, and are an example of a highly heterogeneous sample.^23,31^ The 60-mer AAV capsids (∼3.7 MDa) assemble stochastically from three structurally interchangeable subunit variants of different mass (VP1, VP2, VP3), resulting in an extremely heterogeneous mass distribution of 1891 possible capsid stoichiometries (SI Figures 4 and 5).^23^ Applying eq 2 with the mass and expected charging of such particles, it can be seen that the expected Δmass where peak overlap occurs is in the range of about 21 ± 9 kDa, in which peak overlap will occur at resolution settings reported for experimental spectra of these particles using Orbitrap mass analysers (SI Figure 5).^23^ This mass difference coincides with the mass difference of a single VP1 to VP3 substitution (21.9 kDa for AAV8) explaining the complex interference pattern which adeno-associated viruses display when charged natively (SI Figures 4 and 5).^23^ This complexity precludes the assignment of individual charge states to the overlapping peaks, which is necessary for mass determination.

Expansion of eq 2 to consider overlap of more distant charge states z_*n*_ and z_*n*+*x*_ yields the more general eq 3:
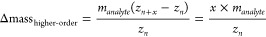
3This shows, for example, that additional overlap of *z*_*n*_ with *z*_*n*+2_ or *z*_*n*+3_ will occur at Δmass two or three times that for z_*n*+1_. This leads to additional overlap at integer multiple Δmass for extremely heterogeneous samples such as AAVs (SI Figure 5C). Therefore, in order to resolve the true charge state distribution of such particles, the peak overlap Δmass threshold has to be increased to much higher values than is possible with chemical charge reductions agents in solution, which only provide moderate charge reduction (see SI Figure 5B). Although deconvolution algorithms and software packages such as MaxEnt and UniDec can facilitate the assignment of complex and even overlapping charge state distributions, cases of extreme charge state overlap, as exhibited by native mass spectra of AAVs, are beyond the deconvolution capabilities of these algorithms.^31−34^ Such heterogeneous analytes have necessitated the development of alternative techniques such as native charge detection mass spectrometry (CDMS), which can simultaneously measure *m*/*z* and charge.^8,35,36^

Alternative methods of charge reduction, involving manipulation of the analyte charge in the gas phase, have the potential to provide greater and more tunable charge reduction; these can involve direct interaction of the ions with free electrons (electron capture) or gas-phase reactions in which electrons or protons are transferred between the protein and an electron donor or proton acceptor reagent.^37−39^ Charge reduction of natively folded proteins was in fact observed initially as an undesirable nondissociative side-reaction of top-down fragmentation experiments using electron capture dissociation (ECD) or electron transfer dissociation (ETD).^38,40−43^ The full mechanistic picture of electron capture/transfer charge reduction has not yet been elucidated, but one idea is that peptide backbone fragmentation does actually occur as a result of the absorbed electron, but the extensive noncovalent interactions in the natively folded, now charged reduced precursor holds the fragments together tightly, such that it can only be dissociated upon further activation.^44−46^ Lermyte et al. showed that ETD could be used to achieve extensive charge reduction of native proteins to ∼120,000 *m*/*z* on a quadrupole-time-of-flight (Q-TOF) mass spectrometer, which is the effective mass range of this particular instrument.^45^ Charge reduction to even higher *m*/*z* scales offers potential for improved resolution of even greater heterogeneity in protein analytes, and can also serve as a test to explore and further develop the high *m*/*z* capabilities of existing instrumentation.

ECD was previously limited to Fourier transform-ion cyclotron resonance (FT-ICR) instruments.^47−50^ The recent development of electron capture cells, which use magnetic fields to trap an electron cloud in the path of the ion beam, made ECD compatible with commercial Orbitrap and Q-TOF instrument platforms and has greatly increased its practicality resulting in a recent flurry of research applying ECD for protein fragmentation and dissociation.^51−64^ The application of this new generation of ECD devices to charge reduction, however, remains neglected. In this work we explored the use of electron capture charge reduction using the ExD TQ-160 (e-MSion Inc.) electron emission cell on the Thermo Scientific Q Exactive UHMR (Ultra-High Mass Range) Orbitrap Mass spectrometer (MS).^51^ The UHMR MS instrument is a popular instrument for native mass spectrometry due to its high resolving power, *m*/*z* range (specified up to 80,000 *m*/*z*) and enhanced activation and desolvation capabilities.^6,65−67^ It has been successfully used for the characterization of megadalton complexes, such as ribosomes, hepatitis B virus capsids (3–4 MDa) and the flock house virus (∼9.3 MDa).^65,68^ Growing interest in the characterization of larger particles such as exosomes, the bacteriophage T5 (∼105 MDa), adenoviruses (up to 156 MDa) and carboxysomes (>300 MDa), which would natively charge (SI Figure 1) above the 350–80,000 *m*/*z* commercial specification of the UHMR MS product line, prompts the need to further explore the high *m*/*z* detection capabilities of existing Orbitrap instrumentation.^69−74^ We therefore set out to explore the use of the e-MSion ExD cell for electron-capture charge reduction (ECCR) on the UHMR with the following goals: (i) examine the tunability of ECCR using the ExD cell, (ii) investigate the instrument parameters affecting the extent of charge reduction, (iii) use the high *m*/*z* ions thus generated to explore the upper *m*/*z* range of the UHMR beyond the commercial specification, and through instrument modification extend the *m*/*z* range if possible, and (iv) explore the application of ECCR for the resolution of overlapping signals in extremely large, heterogeneous analytes.

## Results

### ECCR is Tunable and Achieves up to 90% Charge Reduction of GroEL

**Figure 2:**
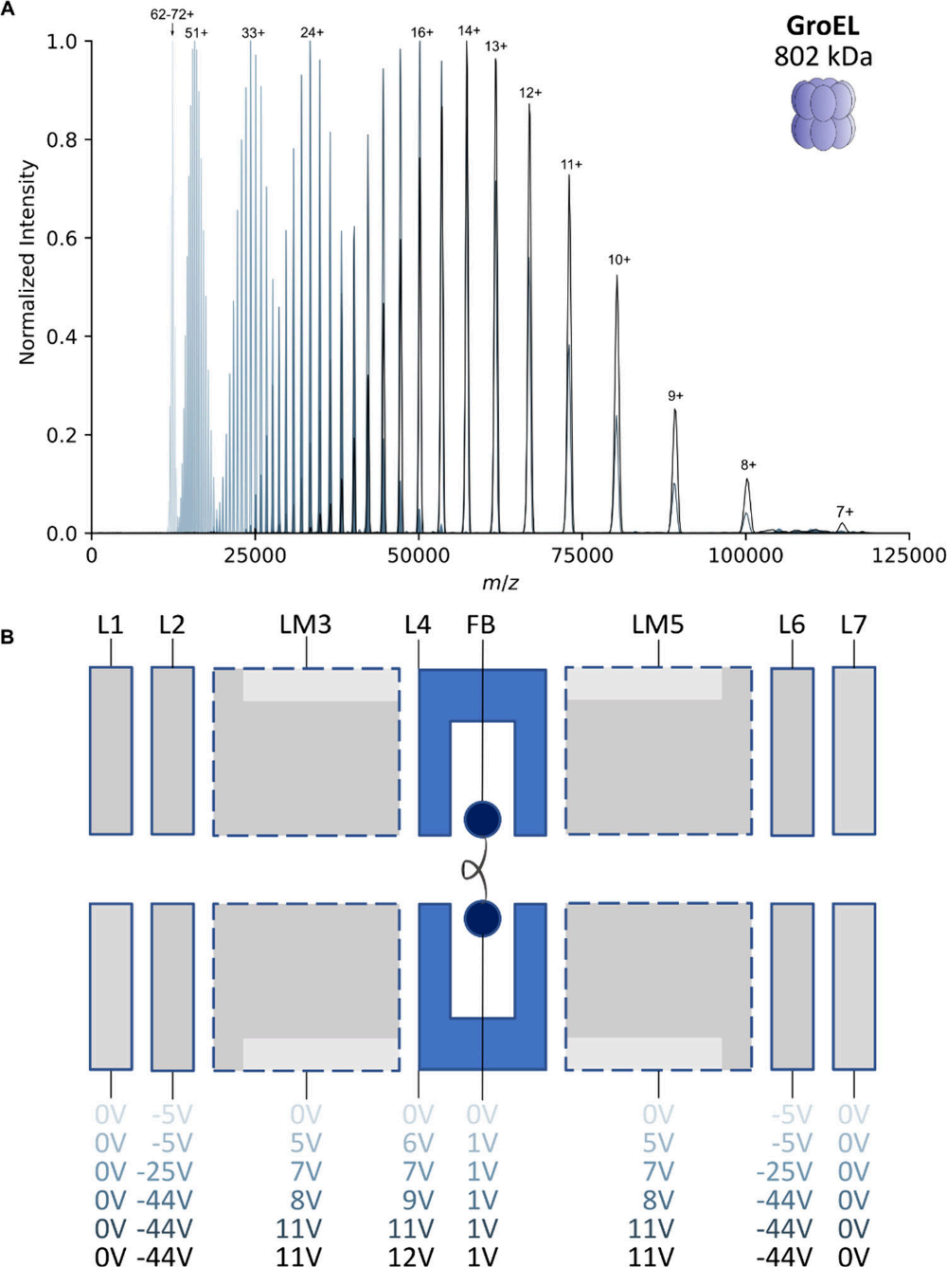
Tunable charge reduction of GroEL, reducing its charge by ∼90%. (A) Native mass spectra of GroEL acquired under a normal native charging regime or with different amounts of electron capture charge reduction. Spectra were acquired with identical UHMR settings and spray conditions but with different voltage settings on the ExD cell. (B) Schematic of the ExD cell comprising lenses (L1–L7), lens magnets (LM3 and LM5), and a metal filament (FB; filament bias voltage). Voltages applied to the elements of the ExD cell in order to obtain the spectra displayed in (A) are shown below the schematic, with colors matching those shown in part (A). For the native charging spectrum (pale blue), the filament current was set to 0 A (i.e., ECD off); for all other spectra, this was set to 2.3 A.

In the modified QExactive UHMR MS instrument, the ExD cell is positioned in the ion beam path after the quadrupole and replaces the transfer multipole (SI Figure 6). It consists of an electron-emitting coiled filament and seven additional lenses and lens magnets along the ion beam path (Figure 2B).^51^ When a current is passed through the filament with a voltage offset applied between it and lens 4, low energy (<3 eV) electrons are emitted and radially confined by the magnetic fields of lens magnets 3 and 5. Application of negative voltages to the outer lenses confines the electrons axially. Electron density is limited primarily by space charge effects from other electrons, and the electrons move rapidly from the filament into the cell. A cloud of low energy electrons is consequently confined along the ion beam path. Tuning of the filament current and ExD cell voltages provides control over the electron capture process by controlling the distribution and density of the electron cloud within the cell, as well as affecting ion trajectories before, during, and after interaction with the electron cloud.

Using the 802 kDa bacterial chaperonin GroEL as a test case, tunable charge reduction over a broad *m*/*z* range was achieved by varying the ExD voltages (Figure 2) with fixed UHMR instrument settings. With the ExD cell tuned to a transmission only mode, without electron emission, GroEL was detected centered on its 65+ charge state at 12,340 *m*/*z*, with an observed native charge state envelop between 59+ and 70+.^75^ −5 V was applied to L2 and L6 during this acquisition to assist with ion transmission. ECCR was initiated by applying the filament current (2.3 A), allowing it to stabilize, then setting the filament bias (FB) to 1 V and increasing the L4 voltage to 6 V in 1 V steps, resulting in electron emission. LM3 and LM5 were increased in small steps to 5 V. These settings resulted in a clear shift of the entire GroEL charge state envelope to the right (Figure 2A).

The amount of charge reduction could be controlled and could be increased by increasing the voltages on L4, LM3, and LM5. It was observed consistently that greater ECCR results in a decrease in the observed total ion signal, which has multiple causes. The intensity of the image current generated on the electrodes by an ion packet is linearly proportional to the total charge of the ion packet. Charge reduction will therefore cause signal intensity loss as a matter of instrumentation principles. Additional losses may arise from perturbation of ion trajectories by the electron cloud, lens voltages, and electron-capture dissociation processes. It is therefore important to try to mitigate the loss of signal by adjusting settings to maximize transmission while also attaining the desired charge reduction. While increasing positive voltages on LM3, L4 and LM5 it was found that larger compensatory negative voltages applied to L2 and L6 were necessary to try to mitigate reduction in the total ion signal. The loss in signal intensity observed during charge reduction of GroEL is shown in SI Figure 3, which plots the relative intensities of the data presented in Figure 2A.

With maximal charge reduction (before reaching the limit of unacceptable signal loss), it was possible to charge reduce GroEL down to the 7+ charge state at 114,600 *m*/*z* (Figure 2A, black spectrum), beyond the commercial specification of 80,000 *m*/*z*, without further instrument modifications. Charge states lower than 6+ were not observed, but charge reduction could be arbitrarily achieved across the entire commercial *m*/*z* range and beyond down to the 7+ charge state through tuning of the ExD cell voltages. These results are in strong agreement with ECCR results for GroEL shown by Shaw, Harvey, Wysocki, and co-workers and others.^76−80^

### Ion Kinetic Energy Affects the Extent of Electron Capture Charge Reduction

**Figure 3:**
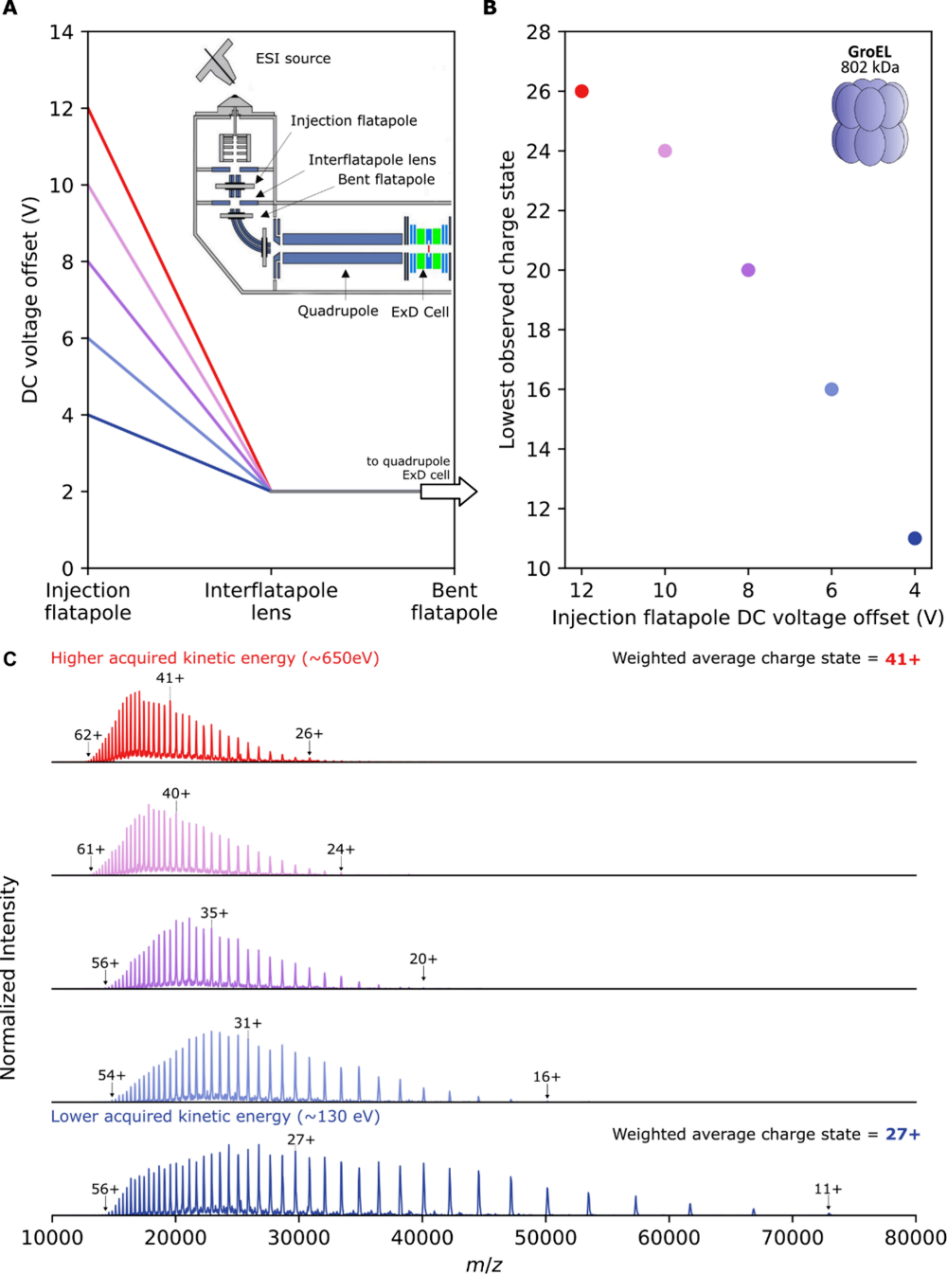
Ion kinetic energy affects the extent of charge reduction. (A) Illustration of how the DC offset voltages of the injection flatapole can be varied (red: 12 V, through to dark blue: 4 V) while maintaining a fixed DC offset voltage of 2 V on the interflatapole lens and bent flatapole in order to control the kinetic energy of the ions as they enter the quadrupole and pass through to the ExD cell. The ions will acquire greater kinetic energy when the voltage offset is higher and move faster through the ExD cell. Inset: schematic of the source and high vacuum region of the UHMR, with relevant ion optics labeled. (B) Lowest detected charge state of GroEL observed during ECCR MS with fixed ExD cell voltages while varying the flatapole voltages as shown in panel (A). (C) Corresponding spectra for the data points in panel (B), obtained with the flatapole settings shown in panel (A).

Variation in UHMR ion optics settings also influences the extent of charge reduction, especially settings that influence ion kinetic energy before the ions reach the ExD cell. This allows further control over the extent of charge reduction by varying the transit time of the analyte ions through the electron cloud. To demonstrate this effect we varied the voltage offset experienced by the ions as they pass from the injection flatapole to the bent flatapole, prior to entry into the quadrupole and ExD cell (Figure 3A). Ions accelerated by a greater voltage drop will acquire a greater kinetic energy. Considering the 65+ charge state of GroEL in the high kinetic energy regime (Figure 3A) with a DC voltage drop from 12 to 2 V we can estimate the upper initial kinetic energy acquired by the ions as 650 eV neglecting collisions, and for the lowest kinetic energy regime (4 to 2 V) as 130 eV, resulting in an up to 5-fold difference between the highest and lowest acquired kinetic energies in the experiment, and consequently up to √5 = 2.24 times different velocities. Maintaining fixed settings on the ExD cell and the rest of the instrument, we consistently found that spectra in the lower kinetic energy regime, when ions are slower, exhibited greater charge reduction (Figure 3B and C). In native top-down ETD experiments using gas phase electron transfer reagents, it has been shown that increasing the reaction time leads to more extensive charge reduction.^46^ Our observation that reducing ion kinetic energy leads to more extensive electron capture charge reduction is consistent with these findings for ETD, as the slower-moving, lower-kinetic energy ions inherently spend more time in the ExD cell interacting with the electron cloud, increasing the opportunity to undergo electron capture events. It is interesting to note that, in contrast to the results of Figure 2A, where tuning ExD voltages to increase charge reduction shifted the entire charge state distribution to lower charge states, tuning of the flatapole voltages instead extends the distribution to lower charge states while maintaining a population of higher charge states. The origin of these distinct behaviors is not clear, but might be related to a larger diversity of pathways and kinetic energies of ions when flatapole acceleration is used. Nevertheless, the finding that control of ion kinetic energy also allows tuning of charge reduction is fruitful, as it provides an additional means to control and achieve more extensive charge reduction in combination with the voltage settings on the ExD cell.

### Simulating the Effect of Charge Reduction on Heterogeneous Adeno-Associated Virus Assemblies

Having demonstrated the feasibility of extensive ECCR on the UHMR instrument (in agreement with related contemporary work^76^) and identified the factors allowing us to control the extent of charge reduction, we next endeavored to investigate its applicability to considerably larger and more heterogeneous analytes. An incidental advantage, when performing ECCR on heavier analytes, is that their larger exposed surface means each particle will acquire a greater number of charges during native nano-ESI,^10^ meaning they will induce greater signal per ion on the Orbitrap detector when charge-reduced to the same *m*/*z* range as a smaller native protein with fewer charges. To this end, we selected AAVs as suitably heavy (∼3.7–4 MDa) and extremely heterogeneous analytes.

**Figure 4:**
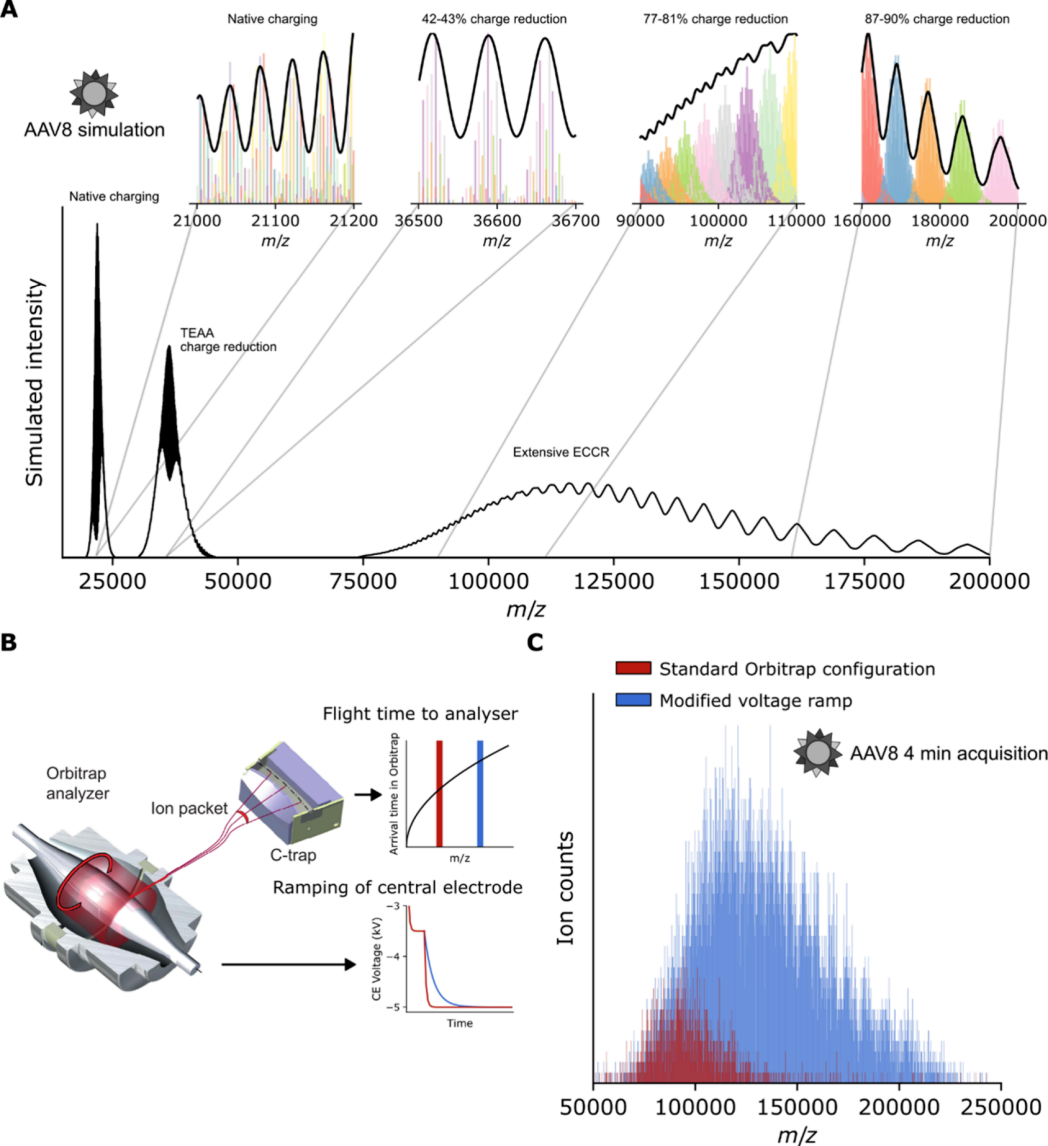
Charge state resolution of AAV8 virus samples should be achievable if charge reduced beyond 100,000 *m***/***z*; modification of the rate of the Orbitrap voltage ramp extends the accessible *m***/***z* range to 200,000 *m***/***z* and beyond. (A) Simulated AAV8 mass spectra assuming stochastic capsid assembly from a pool of VP1, VP2, and VP3 subunits in a 1:1:10 ratio. Simulations are shown for native charging conditions, charge reduction through the use of TEAA, and charge reduction above 100,000 *m*/*z* by ECCR at a resolution setting of 3000. Insets show the individual ion signals (for all 1891 possible capsid stoichiometries) contributing to the simulated peaks in different *m*/*z* regions, with distinct charge states within each inset shown as different colors. Under native charging conditions, the numerous overlapping charge states produce a complex interference pattern. Aligned charge states become resolvable following charge reduction above 100,000 *m*/*z*. (B) Illustration of the time-of-flight effect of ion injection from the C-trap into the Orbitrap analyzer, and ramping of the voltage on the Orbitrap central electrode. Different rates of ramping of the orbitrap electrode (red and blue traces) will result in a different maximum *m*/*z* of ions which can reach the detector in time to adopt stable orbits. (C) Charge-reduced AAV8 mass spectra (short 4 min acquisitions) acquired with the standard UHMR configuration (red) or with a voltage ramp modification (blue) which enhances the detection capability above 100,000 *m*/*z*.

AAV capsids are composed of 60 subunits drawn from three structurally interchangeable proteins of different mass: VP1, VP2 and VP3. The prevailing model of AAV capsid assembly is that they assemble stochastically from the available expression pool of VP1, VP2 and VP3; producing a mixture of capsids of different VP stoichiometry and therefore mass.^23^ As a consequence of this mass heterogeneity, under normal charging conditions, AAV native mass spectra produce an interference pattern from many overlapping signals of different mass and charge (Figure 4A, SI Figures 4 and 5).^23,31^ In the natively charging simulation, there are, for example, up to 12 unique charge states (and multiple masses per charge state) overlapping within a single observed peak. Such a spectrum eludes charge state assignment and, therefore, mass deconvolution. As noted in a recent publication, moderate charge reduction (simulated in Figure 4A), for example, by addition of triethylammonium acetate (TEAA), in fact appears to worsen this problem, producing even greater interference of misaligned charge states.^31^ Our simulations (Figure 4A) predict, however, that when AAVs are extensively charge reduced, the same charge states (of different masses) eventually all begin to group together with sufficient separation from adjacent charge states, such that charge state assignment should become possible beginning from around the 36+ charge state at ∼100,000 *m*/*z*. Correct charge state assignment should then enable calculation of the mean mass of the AAV capsid assemblies, a feat which has previously only been possible by alternative techniques such as charge detection mass spectrometry or mass photometry.^23,81−85^

### Modifying the Rate of the Orbitrap Central Electrode Voltage Ramp Extends the Upper *m*/*z* Range to 200,000 *m*/*z* and Beyond

Following the same methodological principles used to charge reduce GroEL, we were able to charge reduce empty AAV8 capsids to approximately 120,000 *m*/*z* (Figure 4C, red spectrum), demonstrating that it is possible to reach the *m*/*z* region in which simulations indicate it should be possible to achieve charge state resolution. We noted with interest, however, that despite the greater charge (and therefore greater signal intensity) of the AAV8 capsids, the signal intensity faded out around 120,000–130,000 *m*/*z*, just like we observed with GroEL (Figure 2A). This observation points toward instrumentation as the factor limiting the effective *m*/*z* range, especially considering that the maximally charge-reduced spectra are acquired at *m*/*z* values 25% higher than the instrument specification. We therefore sought to increase the detection capabilities of the instrument above 100,000 *m*/*z*.

Injection of ions from the C-Trap into the Orbitrap analyzer is a time-dependent process, with higher *m*/*z* ions arriving at the Orbitrap later than lower *m*/*z* ions (Figure 4B). Meanwhile during injection, the Orbitrap central electrode voltage is ramped down from an initial injection voltage to the final negative measurement voltage.^86^ This ramping toward increasingly negative voltage compresses the ion trajectories, pulling them closer to the central electrode (electrodynamic squeezing), and is essential for the establishment of stable ion trajectories and avoidance of collision with the outer electrodes. Crucially, the timing of the voltage ramp needs to be aligned with the arrival time of the ions in the *m*/*z* range that is desired to be detected.^86^ Slowing the rate of the voltage ramp should increase the upper *m*/*z* limit by allowing more time for higher *m*/*z* ions to arrive at the Orbitrap entrance and adopt stable orbitals by electrodynamic squeezing.

On a research-grade UHMR MS instrument, we made modifications to the Orbitrap electronics to enable switching between the standard configuration and a configuration with a reduced rate of central electrode voltage ramping. With AAV8 charge-reduced as much as could be observed under the standard configuration (Figure 4C, red spectrum), we observed that switching to the modified voltage ramp (Figure 4C, blue spectrum) resulted in a dramatic increase in ion signals above 80,000 *m*/*z*, without a reduction in signal intensity below this threshold. The modified configuration furthermore enables detection of ions at *m*/*z* values which were not detected with the standard configuration, with clear ion signals being detected up to about 250,000 *m*/*z*. These are charge-reduced AAV8 capsid ions which were produced by ECCR and present in the instrument but were simply of too high *m*/*z* to be detected in the standard configuration. Notably, resolved charge states are visible despite the short acquisition time.

### ECCR above 100,000 *m*/*z* Enables Charge State Resolution and Average Mass Determination of Adeno-Associated Virus Assemblies

**Figure 5:**
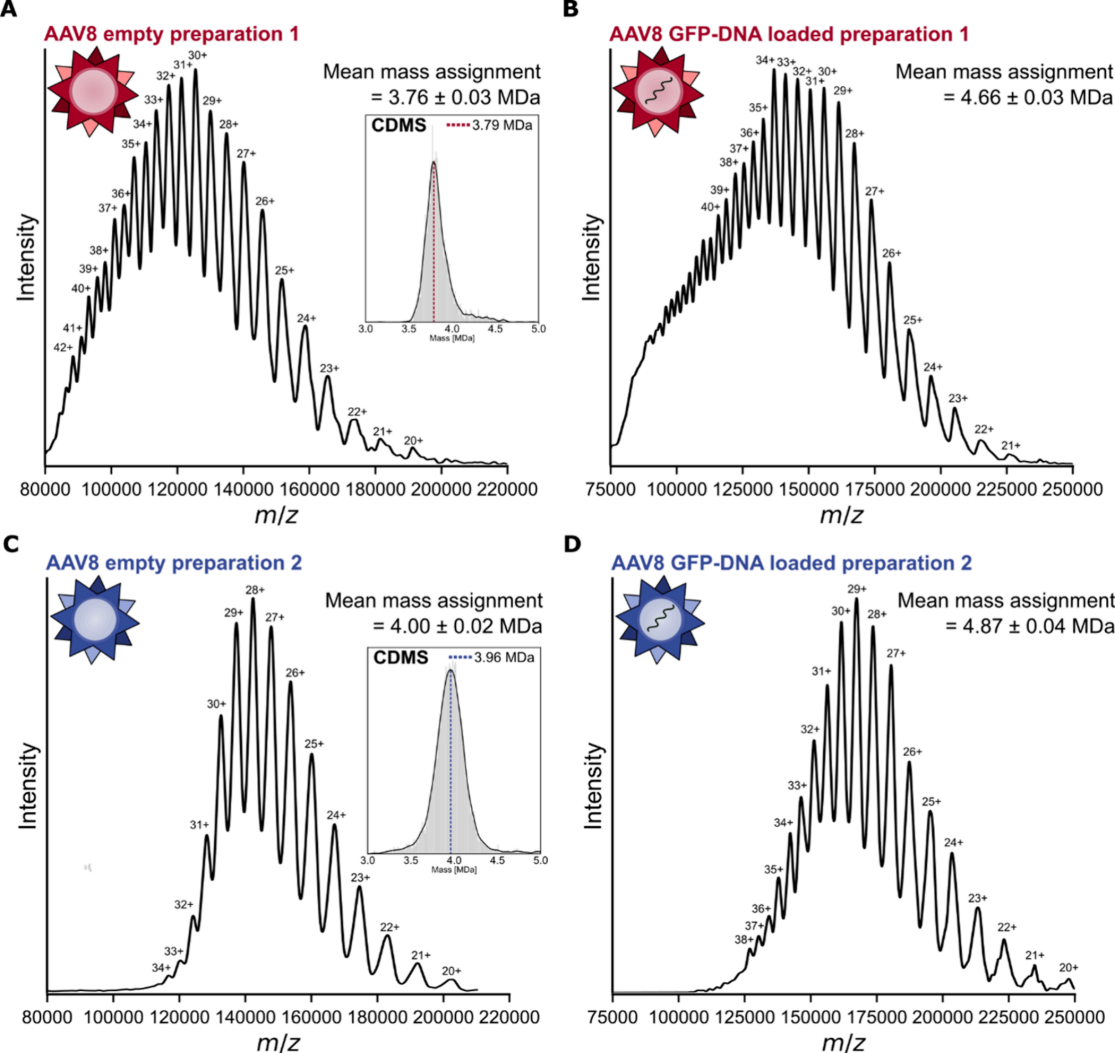
Charge state resolution and mean mass determination of different AAV8 preparations by electron capture charge reduction. Mass spectra of charge-reduced AAV8 from two different preparations enabled charge state assignment and mean mass determination. (A) Preparation 1 empty capsids; inset: charge detection mass spectrum obtained from the same sample. (B) Preparation 1 capsids filled with CMV-GFP genetic cargo. (C) Preparation 2 empty capsids, inset: charge detection mass spectrum obtained from the same sample. (D) Preparation 2 capsids filled with GFP encoding DNA vector.

Long acquisitions with the modified voltage ramp enabled definitive charge state resolution and average mass determination for different AAV8 preparations acquired from different sources (Figure 5). For each preparation, measurements were conducted for both empty capsids and capsids filled with single-stranded DNA cargo encoding green fluorescent protein (GFP). The ECCR mass spectrum for preparation 1 of empty capsids exhibited a broad hump from ∼80,000 *m*/*z* to 200,000 *m*/*z*, bearing peaks to which charge states can unambiguously be assigned using the chevron method (see Materials and Methods).^87^ Calculation of the mass across all assigned charge states yields a mean mass measurement of 3.76 ± 0.03 MDa. This is consistent with a capsid stoichiometry of 1:1:10 (VP1:VP2:VP3; theoretical mass 3.74 MDa) and matches widely reported mass measurements for commercially available AAV8 preparations using alternative techniques, such as CDMS and mass photometry, confirming the correct charge state assignment.^8,84^ For further confirmation of the mass, we performed CDMS on the same sample (Figure 5A inset). We observed consistent mass differences across the charge states within each of the ECCR spectra, arising from reduced desolvation of the more charge-reduced ions; such ions experience reduced acceleration and therefore less collisional desolvation in the HCD cell. This mass difference assists in charge state assignment, the correct set of charge assignments being that which satisfies two constraints: (i) minimization of the standard deviation in mass across the charge state distribution and (ii) an inverse correlation between charge and mass (SI Figure 7). The deconvolution of the overlapping charge state distribution of extremely heterogeneous AAV assemblies by native mass spectrometry is an exciting outcome, with promising application to other heterogeneous and stochastically assembling analytes.^31^ AAV8 capsids loaded with genetic material could also be charged state resolved by ECCR (Figure 5B). This is surprising, due the additional mass and heterogeneity added by the genetic vector, which can consist of either sense or antisense DNA. The mean mass difference between the full and empty particles points toward a genome mass of 0.900 ± 0.046 MDa, again consistent with literature and the diverse masses of such megadalton particles.^8^

AAV preparations from different suppliers and expression systems are known to exhibit variation in stoichiometry and genome packaging and therefore in mass.^31,84^ We therefore performed ECCR on a different preparation (preparation 2) of the same AAV8 serotype, again with empty capsids and capsids filled with genetic material (Figure 5C and D). These samples were charge reduced slightly more than the samples for preparation 1 under similar conditions, and once again, ECCR yielded charge state resolution and enabled assignment. These spectra yielded a higher than expected mass for empty AAV8 (4.00 ± 0.02 MDa measured vs 3.74 MDa theoretical mass assuming 1:1:10 VP1:VP2:VP3 ratio), but the greater mass is supported by CDMS (Figure 5C inset) and a genome mass of 0.87 ± 0.04 MDa determined by comparison of the full and empty spectra. The additional mass may be due to a higher than expected VP1:VP2:VP3 ratio or unexpected encapsulations.

The *m*/*z* range and charge reduction displayed in these spectra are remarkable. With ions detected up to 250,000 *m*/*z*, these represent the highest *m*/*z* ions ever detected on an Orbitrap mass spectrometer; furthermore, the ions were detected over an *m*/*z* span of ∼150,000 *m*/*z*, which is almost twice as large as the upper *m*/*z* commercial specification of the UHMR.

## Discussion and Conclusions

This work demonstrates that controllable electron capture charge reduction can be achieved over a broad *m*/*z* range on the Q Exactive UHMR mass spectrometer, with the charge-state resolution and therefore mean mass determination of highly heterogeneous stochastically assembling adeno-associated virus capsids as a capstone application. Charge-state assignment of AAVs had until now been impossible by conventional native mass spectrometry, and this had stimulated the development of alternative techniques such as charge detection mass spectrometry.^8,35,81^ Compared with charge detection mass spectrometry, charge state resolution via ECCR has the advantage of more confident charge state determination and has less stringent requirements in terms of Orbitrap gas pressure or ion stability than CDMS. It has recently been highlighted that beyond AAVs, stochastic assembly of macromolecular complexes may be more common than previously realized; a phenomenon which has been overlooked and can lead to erroneous charge assignments.^31^ While limited charge reduction can potentially worsen the spectral appearance of such spectra, our work shows that extensive charge reduction presents an elegant and straightforward solution even for extreme cases such as AAVs. Given the increasing use of viral and lipid nanoparticle-based delivery systems for vaccination and gene therapy, ECCR presents new possibilities for characterizing these often highly heterogeneous systems.^88−90^

Decreased desolvation of extensively charge-reduced species due to their lesser acceleration by electric fields is a major factor limiting resolution in these experiments however. Combining ECCR with charge state independent methods of desolvation, such as IR photoactivation, could greatly enhance the power of this technique and is a promising direction for future research.^91,92^ In the fields of gas-phase chemistry and electron capture/transfer dissociation, it is remarkable that viral capsids in the gas phase can capture over 100 electrons and that the increasingly charge-deprived particles are able to continue to react effectively with more electrons within the time frame of the ion/electron interaction, even where ∼90% of initial charge is neutralized. The charge reduced ions survive intact to the detector, without apparent dissociation, consistent with nondissociative electron capture observed for smaller protein ions in previous studies.^37,48,49,87^

This work further represents a technical advancement in Orbitrap instrumentation. Without modification, we have shown that the UHMR can transmit and detect ions up to ∼130,000 *m*/*z*; 50,000 *m*/*z* higher than its 80,000 *m*/*z* commercial specification. With modifications, we were able to detect ion signals at ∼250,000 *m/z*, the highest *m*/*z* ions recorded to date on an Orbitrap mass spectrometer. This positions Orbitrap native mass spectrometry well in the context of growing interest in the characterization of larger particles such as exosomes, adenoviruses and carboxysomes.^69−72^ The 156 MDa human adenovirus 5, for example, has been observed with charge states around 1100+ placing it at ∼142,000 *m*/*z*, well within the detection capability of our modified UHMR.^71^ At this *m*/*z* range, adenovirus would have approximately 45 times the number of charges than the 24+ charge state of adeno-associated viruses which appear in the same *m*/*z* region; the 156 MDa adenovirus would therefore produce ∼45 times more signal and be even easier to detect than the charge-reduced AAV ions detected in this work. With the *m*/*z* range demonstrated by our modified UHMR, detection of ions up to 550 MDa in mass is theoretically possible assuming native charging behavior (SI Figure 1).

The high *m*/*z* region and extensive charge reduction to this region present a number of technically interesting features and applications. High *m*/*z* ions, having lower frequencies, travel reduced distances in the Orbitrap analyzer at lower velocities compared to lower *m*/*z* ions over the same transient time (SI Figure 8), resulting in fewer and less energetic collisions and hence more stable trajectories.^68^ This has potential applications for use cases such as Orbitrap CDMS and ultralong transient acquisitions, for which ion stability is a key factor in the quality of results. We have furthermore shown how extensive and tunable charge reduction to an *m*/*z* range of choice can serve as a useful tool in instrumentation development, allowing the assessment of instrument capabilities over its entire *m*/*z* range. The ability to generate ions at controllable *m*/*z* furthermore has potential application for *m*/*z* calibration and for charge detection calibration in CDMS.

Various terminologies have been used to describe the charge reducing effect of electron and proton capture/transfer, such as ECnoD (electron capture no dissociation), charge reduction ETD, and proton capture charge reduction. More recently the term ECCR has been used.^76,77,79,80^ Electron transfer charge reduction (ETCR) and proton transfer charge reduction (PTCR) also naturally follow this terminology. We propose that the research community adopts this style of terminology when these techniques are used for charge reduction applications, where fragmentation is not the objective. These acronyms and terminology make clear which type of charge reduction process is being employed and distinguish this application from fragmentation experiments involving ECD and ETD. We expect that the utility and general methodology of native charge reduction described in our paper will also extend to charge reduction achieved by ETnoD and PTCR, and will furthermore be generally applicable to different types of ExD devices and mass spectrometers.

Given the exciting results demonstrated here, and the present practical availability of devices for generating and confining free electrons within commercial mass spectrometers, we believe that ECCR is set to enable exciting progress in the separation of overlapping charge states in native mass spectrometry, especially when combined with additional emerging techniques such as charge detection mass spectrometry, ultralong transients, and surface induced dissociation.^76,85,93^

## Materials and Methods

### Preparation of Protein Samples for Native MS

Recombinant *E. coli* chaperonin GroEL was purchased from Sigma-Aldrich. For purification the lyophilized powder was dissolved at a concentration of 1 mg/mL in 1 mL of reconstitution buffer (20 mM tris acetate, 50 mM potassium chloride, 0.5 mM EDTA, 1 mM ATP and 5 mM MgCl_2_) and vortexed for 1 h at room temperature. 200 μL of ice cooled methanol was added, and the solution was vortexed again for 1 h at room temperature. Assembled GroEL oligomer was precipitated by addition of 600 μL ice cooled acetone and left for 4 h at 5 °C. The supernatant was removed and the precipitate redissolved in the reconstitution buffer. For native MS application, GroEL was buffer exchanged into ammonium acetate (50 mM) by overnight dialysis at 5 °C and used for native MS at ∼5 μM final concentration.

Preparation 1 empty and full (filled with CMV-GFP genetic cargo) AAV8 capsids were purchased from Virovek. Preparation 2 empty and full (filled with ZsGreen GFP genetic cargo) AAV8 capsids were expressed and purified as described below. All AAV8 samples were buffer exchanged into ammonium acetate (50 mM) by overnight dialysis at 5 °C and used for native MS at a final concentration of ∼100 nM final concentration.

### AAV8 Expression and Purification

HEK Viral Production cells (ThermoFisher) were maintained in suspension in Freestyle F17 (ThermoFisher) chemically defined, serum-free media supplemented with 1× Glutamax (Gibco). The cells were cultured in 2 L roller bottles (Greiner) at 37 °C, 7% CO_2_ and 135 rpm agitation in a humidified atmosphere. The plasmids used were pAAV_zsGreen, pAAV_RC8, and pAAV_Helper (produced in-house). Cells were seeded into 9 L of medium at 0.5 × 10^6^ cells per mL in a wave bioreactor (Cytiva) on a Wave25 platform. At 24 h postinoculation, the culture was triple transfected using PEIMax (Polyplus) and three plasmids at a ratio of 2:1.5:1. To produce empty AAV particles, the cultures were transfected identically except with the absence of the transgene plasmid (pAAV_zsGreen).

Each culture was harvested by batch centrifugation (3800*g* for 15 min at 4 °C) 72 h post transfection to separate the cell fraction from the supernatant. The resulting cell pellet was freeze–thawed thrice and resuspended to 10% (w/v) in a lysis buffer containing 5% (v/v) Polysorbate 80 and 20 U mL^–1^ benzonase (Merck). After 1 h of incubation at 37 °C, the lysate was clarified by depth filtration followed by sequential filtration through 0.8 and 0.45 μm filters. The clarified lysate was pooled with filtered supernatant and concentrated by TFF on an Ultracel Pellicon 2, 300 kDa MWCO cassette (Merck) before loading onto a pre-equilibrated POROS CaptureSelect AAVX affinity column (Thermo Fisher) at 1 × 10^13^ ng/mL of resin. After re-equilibration, AAV was eluted with a low-pH buffer and immediately neutralized with 1 M tris base. Neutralised eluate was concentrated and buffer exchanged into PBS using an Amicon Ultra, 100 kDa MWCO concentrator (Merck) before storage at −80 °C.

### VLP Expression and Purification

HEK Viral Production cells (ThermoFisher) were maintained in suspension in Freestyle F17 (ThermoFisher) chemically defined, serum-free media supplemented with 1× Glutamax (Gibco). The cells were cultured in 2 L roller bottles (Greiner) at 37 °C, 7% CO_2_ and 135 rpm agitation in a humidified atmosphere. The plasmids used were pAAV_zsGreen, pAAV_RC8, and pAAV_Helper (produced in-house at AstraZeneca). Cells were seeded into 9 L of medium at 0.5 × 10^6^ cells per mL in a bioreactor (Cytiva Wave25). AT 24 h postinoculation, the culture was triple transfected using PEIMax (Polyplus) and three plasmids at a ratio of 2:1.5:1. To produce empty AAV particles, the culture was transfected identically, except with the absence of the transgene plasmid.

Each culture was harvested by batch centrifugation (3800g for 15 min at 4 °C) 96 h post transfection to separate the cell fraction from the supernatant. The resulting cell pellet was freeze–thawed three times and resuspended to 10% (w/v) in a lysis buffer containing 5% (v/v) Polysorbate 80 and 20 U mL^–1^ benzonase (Merck). After 1 h incubation at 37 °C, the lysate was clarified by depth filtration followed by sequential filtration through 0.8 and 0.45 μm filters. The clarified lysate was pooled with filtered supernatant and concentrated by TFF on an Ultracel Pellicon 2, 300 kDa MWCO cassette (Merck) before loading onto a pre-equilibrated POROS CaptureSelect AAVX affinity column (Thermo Fisher) at 1 × 10^13^ viral genomes per mL (vg/mL) of resin. After re-equilibration, AAV was eluted with a low-pH buffer and immediately neutralized with 1 M tris base. To enrich for full capsids, neutralized affinity eluate was diluted with a low conductivity buffer and loaded onto a pre-equilibrated Capto Q column. The column was washed with a low salt buffer before the elution of full capsids with a high salt buffer. The resulting eluate was buffer exchanged into PBS using an Amicon Ultra, 100 kDa MWCO concentrator (Merck) before storage at −80 °C.

### Native MS Instrumentation

ECCR spectra were acquired on a modified, research grade Q Exactive-UHMR instrument. The instrument was fitted with an ExD TQ-160 (e-MSion) electron emission cell, replacing the transfer multipole. The detector pulser board was physically modified so that the rate of the voltage ramp on the Orbitrap central electrode could be switched from the standard factory configuration to a modified configuration with reduced slew rate. 3 μL aliquots of sample were injected into gold and palladium coated borosilicate glass capillaries, prepared in-house with a model P-97 micropipette puller (Sutter Instrument Company) and sprayed into the instrument by nanoelectrospray ionization.

### Native MS and ECCR of GroEL

The effect of ExD cell settings on ECCR was assessed with fixed DC voltage offsets of 5 V on the injection flatapole, 4 V on the interflatapole lens, and 2 V on the bent flatapole, while varying voltages on the ExD cell as shown in Figure 2B. For the native charging experiment, with the ExD cell in transmission only mode, the filament current was set to 0 A, for all other spectra this was set to 2.3 A. Data were acquired from the same sample-loaded nanoESI capillary, which sprayed continuously during the experiments.

The effect of ion kinetic energy on ECCR was assessed while maintaining a fixed set of ExD cell voltages (L1 = 0, L2 = −27, LM3 = 6.5, L4 = 6.5, FB = 1, LM5 = 6.5, L6 = −27, and L7 = 0) and 2.3 A filament current. The interflatapole lens (2 V) and bent flatapole (2 V) were held at fixed DC offset voltages, while the injection flatapole lens was decreased from 12 to 4 V in 2 V every 5 min as shown in Figure 3. Data were acquired from the same sample-loaded nanoESI capillary, which sprayed continuously during the experiments.

Spectra were acquired by using in-source trapping (IST, −100 V) without HCD cell activation. N_2_ was used as the trapping buffer gas with typical UHV pressure reading of 5.5 × 10^–10^ mbar. High *m*/*z* ion transfer target and high *m*/*z* detector optimization settings were used with the standard (unmodified) configuration of the Orbitrap voltage ramp rate.

### Native MS and ECCR of AAV8 Preparations

AAV8 samples in 50 mM ammonium acetate were spiked with 25 mM triethylammonium acetate immediately prior to nanoESI in order to achieve limited chemical charge reduction prior to ECCR. The preceding chemical charge reduction helped in achieving greater overall maximal charge reduction. To optimize charge reduction, desolvation and signal quality, ExD cell voltages and UHMR ion transfer and desolvation parameters were tuned for each sample and are presented in SI Table S1.

Xenon was used as a collision gas in the HCD cell, with an exemplary UHV vacuum pressure readout of 7.7 × 10^–10^ mbar. Ion transfer and detector optimizations settings were set to high *m*/*z*. Spectra were recorded with a 4096 ms transient time using magnitude mode Fourier transform with FFT enabled. Ions were detected as individual particles, similar to a CDMS experiment, due to low sample concentration, transmission losses from ECCR and the heterogeneity of AAV8 assemblies.^8^

For the experiments shown in Figure 3B, which assess the effect of the Orbitrap voltage ramp rate on detection of ultrahigh *m*/*z* species above 100,000 *m*/*z*, AAV8 preparation 1 empty was charge reduced as much as possible without unacceptable loss of ion transmission and a spectrum was acquired for four min using the standard Orbitrap configuration. The Orbitrap configuration was then rapidly switched to the modified configuration with reduced voltage ramp rate and electrospray was restarted from the same needle, in the same position, with all other instrument settings the same and acquired again for 4 min. The results were reproducible when switching back to the standard configuration. All other AAV8 spectra were recorded using the modified Orbitrap configuration and acquired for 1–5 h depending on spray stability.

### Analysis of AAV8 ECCR Mass Spectra

Raw acquisition files were converted to mzXML file format using the MSConvert tool and vendor peak picking algorithm within the ProteoWizard Toolkit.^94^ The mzXML files were analyzed using python scripts making use of the Pyteomics, NumPy, pandas, SciPy and Matplotlib packages.^95−100^ Electrical noise signals that were persistent across many scans were manually identified and removed, and a custom decaying noise filter function, which accounts for the decreasing intensity of lower charged ions, was used to filter baseline noise. A histogram with 5 *m*/*z* bin spacing was constructed from the filtered ion signals, smoothened by Savitzky-Golay filtering and peaks were picked using the peak picking function of SciPy.^99^ Charge states were assigned using the chevron method, to find the charge assignment which is closest to the minimum of the standard deviation of mean mass calculated across all peaks, and which correctly exhibits differences in desolvation at different charge states (lower charge state peaks should have mass > mean mass; higher charge state peaks should have mass < mean mass), as shown in SI Figure 7.^87^ The correct charge state assignment was always found to be one charge higher than that which globally minimized the standard deviation, as this assignment produces the expected differential desolvation behavior and is consistent with the results of CDMS.

### Charge Detection MS

CDMS spectra were acquired on a standard Q Exactive-UHMR instrument (ThermoFisher Scientific), without modification, following published protocol and analysis scripts.^8^ The charge-intensity constant was calibrated by using GroEL as a reference calibrant. Transients were recorded in enhanced Fourier transform mode at 50k and 100k resolution settings and with 800 ms injection time.

### Simulation of AAV8 Charge Reduction

Published python scripts for the simulation of stochastic assembly of AAV capsids and the resulting heterogeneous native mass spectrum were adapted so that the spectrum could be simulated at different levels of charge reduction, and modeling the decreased desolvation of charge-reduced species.^23^ The simulations furthermore model the expected *m*/*z* dependency of the resolution in an Orbitrap instrument. Simulations were performed for a VP1:VP2:VP3 expression ratio of 1:1:10 with masses 81667.3 Da (VP1), 66518.6 Da (VP2), and 59763.1 Da (VP3).

## Supporting Information Available

UHMR and ExD cell tuning parameters used for AAV8 ECCR mass spectra; expected average *m*/*z* position of biomolecular analytes assuming native charging and near-spherical shape; supplement to Figure 1; supplement to Figure 2; native mass spectrum of empty AAV8 capsids without charge reduction; simulated mass distribution, peak overlap threshold for adjacent charge states, and native mass spectrum of AAV8 capsid assemblies; instrument schematic; charge state and mass assignment for AAV empty ECCR native mass spectra; distance traveled vs *m*/*z* for ions during a 1 s transient; supplementary references (PDF)
Transparent Peer Review report available (PDF)
