# Supplementary material for: To 200,000 m/z and Beyond: Native Electron Capture Charge Reduction Mass Spectrometry Deconvolves Heterogeneous Signals in Large Biopharmaceutical Analytes
Source: ACS Cent Sci. 2024 Jul 26;10(8):1548–61. doi: 10.1021/acscentsci.4c00462 (PMC11363327; doi:10.1021/acscentsci.4c00462)
Supplement: Supplementary file 1 — oc4c00462_si_001.pdf [file oc4c00462_si_001.pdf]

Supplementary Information

To 200,000 m/z and Beyond: Native Electron Capture Charge Reduction Mass Spectrometry Deconvolves Heterogeneous Signals in Large Biopharmaceutical Analytes

Kyle I. P. Le Huray[1] †, Tobias P. Wörner[2] †, Tiago Moreira[1], Marcin Dembek[3], Maria Reinhardt-Szyba[2], Paul W.A. Devine[4], Nicholas J. Bond[4], Kyle L. Fort[2,5] *, Alexander A. Makarov[2,5] * and Frank Sobott[1] *

† These authors contributed equally

[1] Astbury Centre for Structural Molecular Biology, School of Molecular and Cellular Biology, Faculty of Biological Sciences, University of Leeds, Woodhouse Lane, Leeds LS2 9JT, U.K.

[2] Thermo Fisher Scientific (Bremen) GmbH, Hanna-Kunath Str. 11, 28199 Bremen, Germany

[3] Purification Process Sciences, Biopharmaceutical Development, Biopharmaceuticals R & D, AstraZeneca, 1 Francis Crick Avenue, Cambridge CB2 0AA, U.K.

[4] Analytical Sciences, Biopharmaceutical Development, Biopharmaceuticals R & D, AstraZeneca, 1 Francis Crick Avenue, Cambridge CB2 0AA, U.K.

[5] Biomolecular Mass Spectrometry and Proteomics, Bijvoet Centre for Biomolecular Research and Utrecht Institute for Pharmaceutical Sciences, Utrecht University, Padualaan 8, 3584 CH Utrecht, The Netherlands

*kyle.fort@thermofisher.com

*alexander.makarov@thermofisher.com

*f.sobott@leeds.ac.uk

UHMR and ExD cell tuning parameters	AAV8 preparation 1 empty	AAV8 preparation 1 filled	AAV8 preparation 2 empty	AAV8 preparation 2 filled
Filament current	2.3 A	2.3 A	2.3 A	2.3 A
L1 voltage	0 V	0 V	0 V	0 V
L2 voltage	-36.2 V	-52.7 V	-26 V	-58.3 V
LM3 voltage	5.7 V	5.7 V	5.6 V	5.7 V
L4 voltage	6.1 V	6.7 V	6 V	7.1 V
FB voltage	1.0 V	1.0 V	1.0 V	1.0 V
LM5 voltage	5.7 V	5.7 V	5.6 V	5.7 V
L6 voltage	-36.2 V	-52.7 V	-26 V	-58.3 V
L7 voltage	0 V	0 V	0 V	0 V
In-source trapping voltage	-135 V	-150 V	-120 V	-150 V
HCD energy	212 V	212 V	125 V	185 V
Injection flatapole DC offset	3.5 V	3.5 V	4 V	4 V
Interflatapole lens DC offset	2.5 V	3 V	3 V	3 V
Bent flatapole lens DC offset	2.0 V	2.0 V	2.5 V	2.5 V

Suppl. Table S1. UHMR and ExD cell tuning parameters used for acquisition of AAV8 ECCR mass spectra.

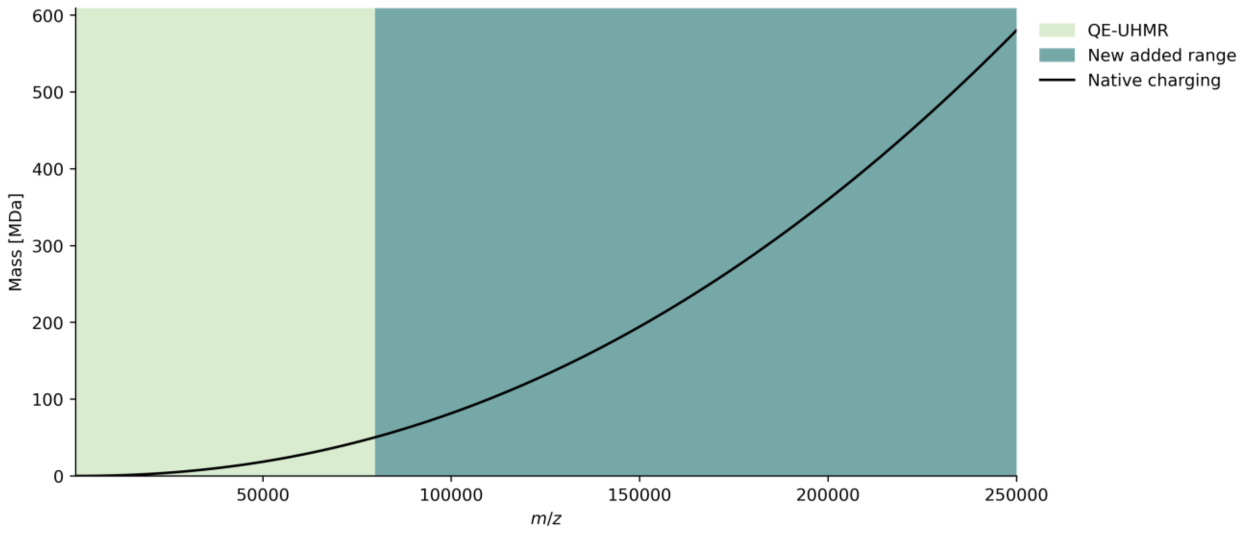

Suppl. Fig. 1. *Expected average m/z position of biomolecular analytes assuming native charging and near-spherical shape; on the basis of empirical equation* $mass = 1.63 \times 10^{-3} \times m/z^{2.14}$ (mass in Da) *observed by Heck and colleagues for analytes up to 20 MDa, and consistent with observations of Jarrold and co-workers for ~156 MDa adenoviruses (1-4). The plot background is coloured to illustrate the standard m/z range of the QExactive UHMR MS and the further range newly added in this work.*

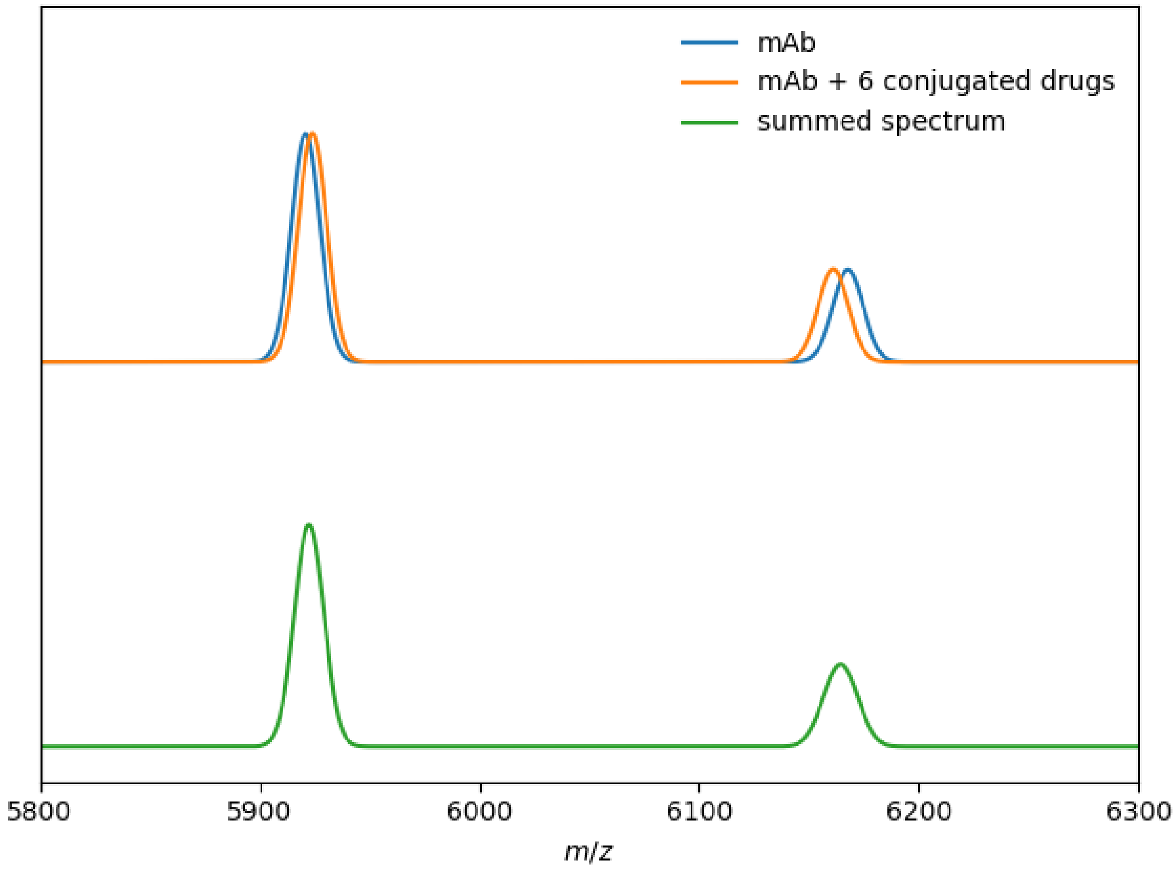

Suppl. Fig. 2. (Supplement to Fig. 1). *Simulated peak overlap between the 25+/26+ (signals around 5920 m/z), and 24+/25+ (~6160 m/z) charge states of a monoclonal antibody (mass = 148 kDa) and its 6 drug conjugated form (mass = 154 kDa) at an Orbitrap resolution setting of 1500.*

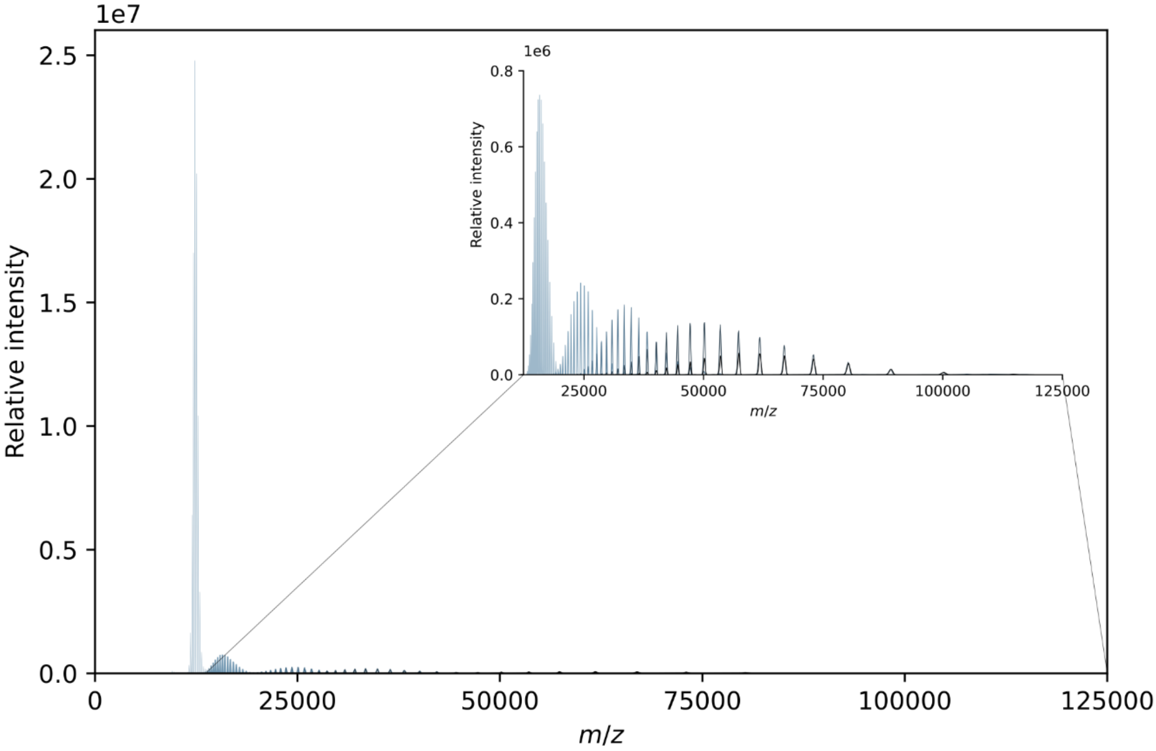

Suppl. Fig 3. *Supplement to Figure 2. Tunable charge reduction of GroEL, reducing its charge by up to ~90%. Native mass spectra of GroEL acquired under a normal, native charging regime (leftmost distribution) or with different amounts of electron capture charge reduction (see inset). Spectra were acquired with identical UHMR settings and spray conditions, but with different voltage settings on the ExD cell. Spectra are plotted scaled to the same relative intensity, to demonstrate the decrease in signal intensity with increasing charge reduction.*

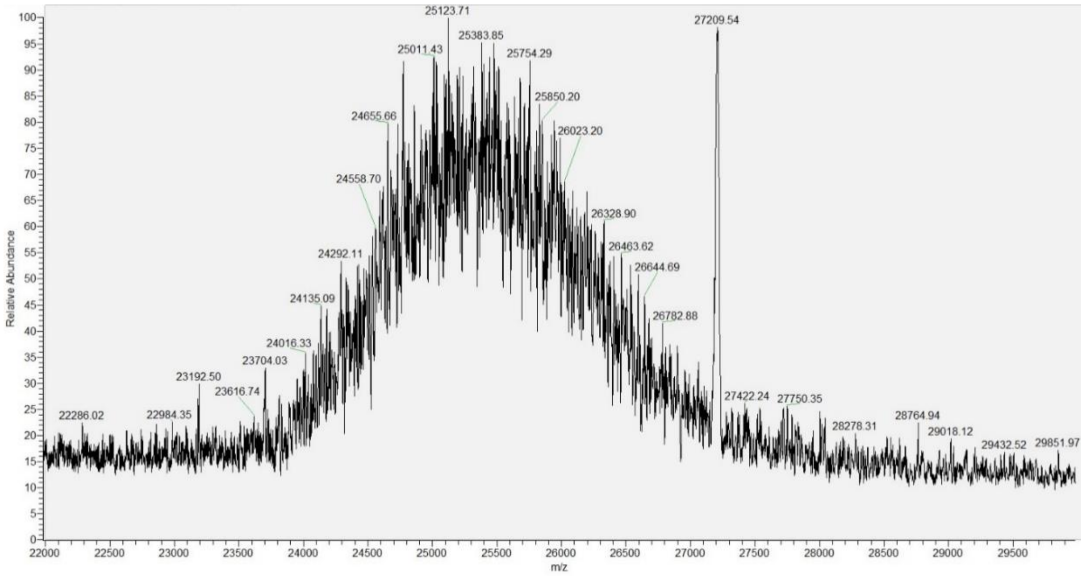

Suppl. Fig 4. *Native mass spectrum of empty AAV8 capsids (preparation 2), recorded with a transient time of 256 ms and without charge reduction. Note that there is a noise peak originating from electrical noise at 27209.54 m/z. As previously shown, and as simulated in Suppl. Fig. 5, this spectrum is an interference pattern resulting from coincidence overlap of signals from many ions of different mass and charge, precluding charge state assignment (5).*

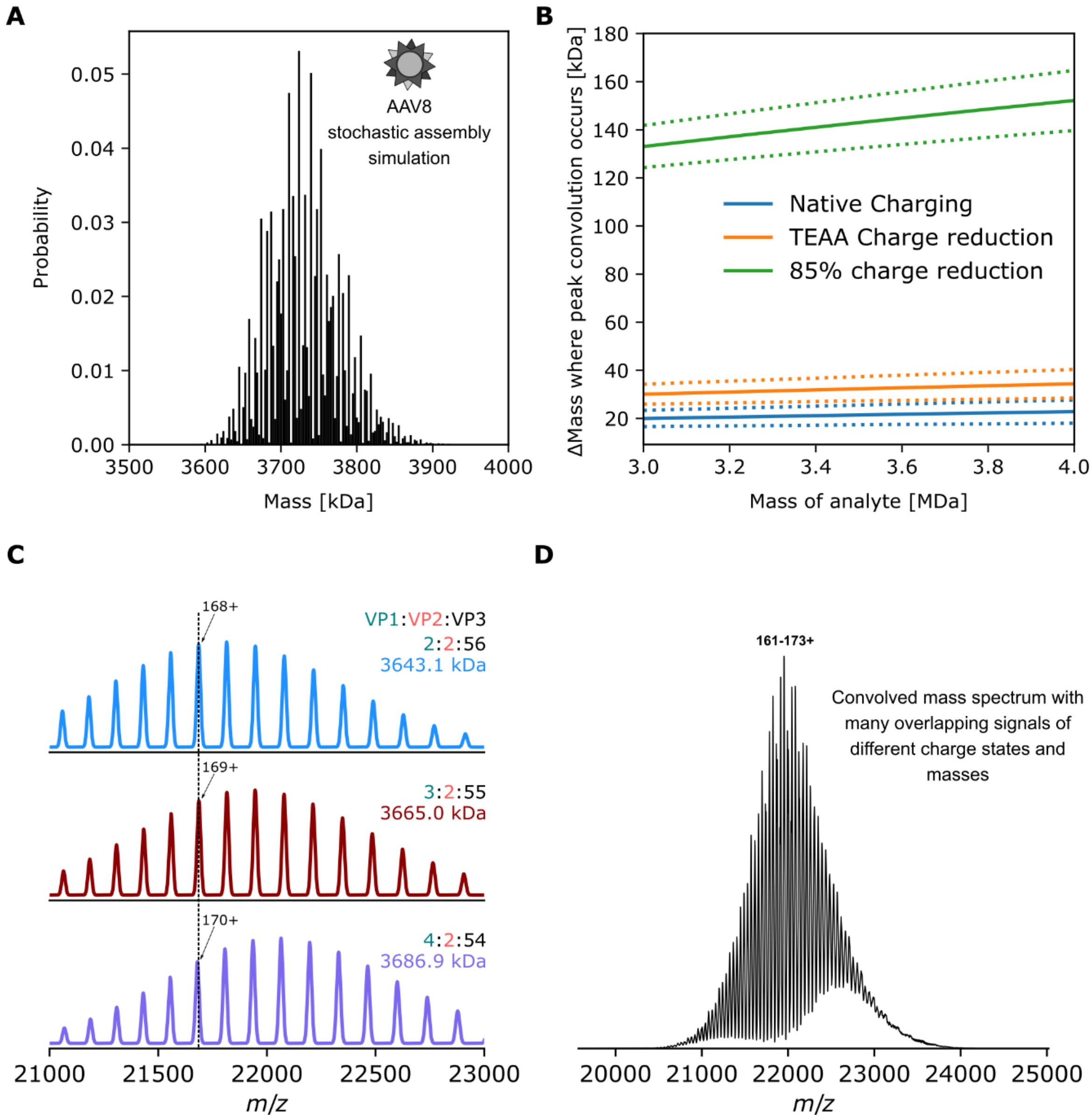

Suppl. Fig. 5. *Simulated mass distribution, peak overlap threshold for adjacent charge states, and native mass spectrum of AAV8 capsid assemblies.* ***A)*** *probability distribution for 1891 possible capsid masses, assuming each capsid is assembled by 60 random draws from a subunit pool of VP1 (81667.3 Da), VP2 (66518.6 Da) and VP3 (59763.1 Da) in a VP1:VP2:VP3 population ratio of 1:1:10.* ***B)*** *Δmass of modifications for which there will be overlap between the z+1 charge state of (analyte mass + Δmass) and the z+ charge state of analyte mass, on the basis of equation 2 and assuming native average charging behaviour (blue), moderate charge reduction with TEAA (orange) or 85% charge reduction (green). Solid lines indicate the Δmass where exact signal overlap will arise, dotted lines bound a window of unresolvable peak overlap due to instrument limitations (here modelled for an Orbitrap transient length of 32 ms, as reported in the literature (5).* ***C)*** *Simulated native mass spectra for three pure capsid masses, illustrating coinciding signals of different charge and mass.* ***D)*** *Simulated native mass spectrum of the mixed capsid assemblies, with mass populations as shown in panel A. The overlap of many signals of different charge and mass produces a complex interference pattern.*

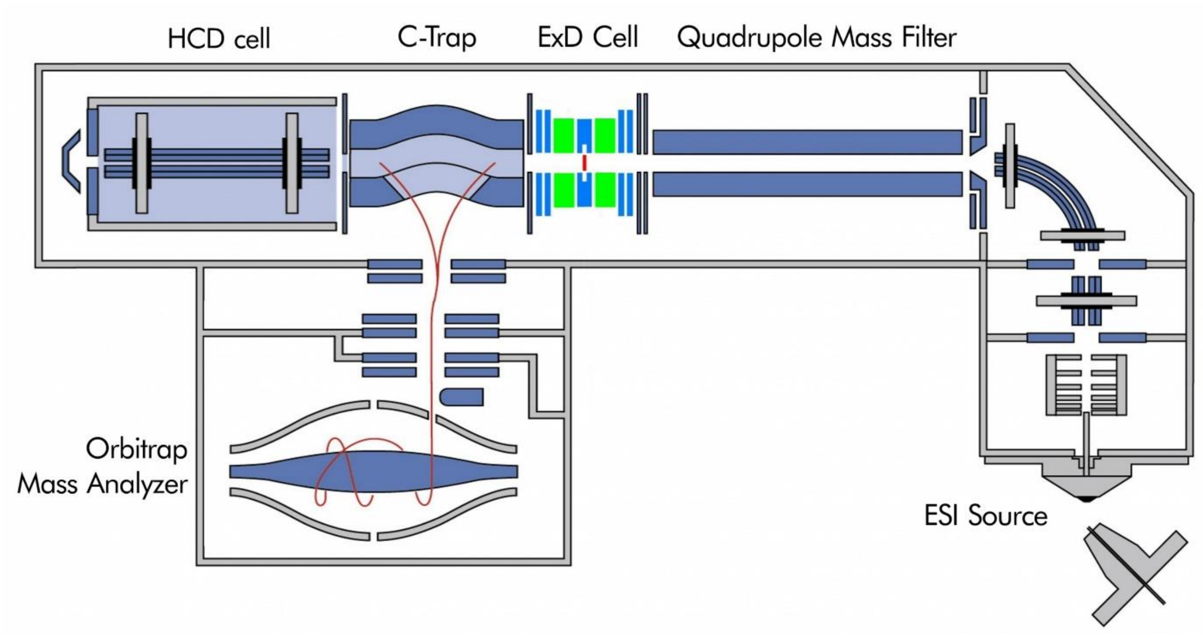

Suppl. Fig. 6. *Schematic of the Q Exactive UHMR MS with the installed ExD TQ-160 cell. The ExD cell replaces the transfer multipole in this instrument.*

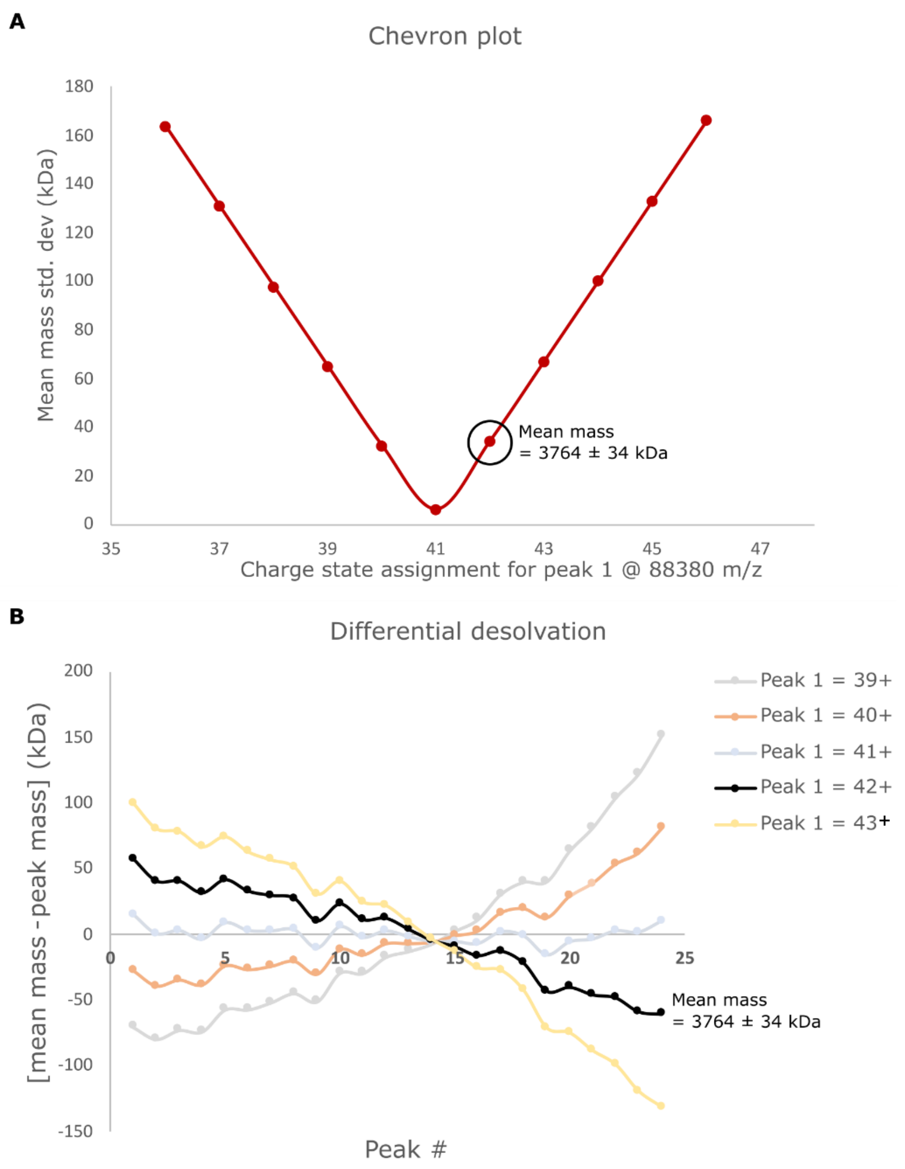

Suppl. Fig 7. *Charge state and mass assignment for AAV empty capsid (preparation 1) native mass spectra with ECCR. Due to extensive charge reduction of the megadalton sized AAV particles, there will be an apparent mass shift across the charge states due to differential desvolation. The lower charged ions will experience reduced acceleration and collisional activation in the HCD cell, and will therefore retain greater residual solvation. This mass shift must be taken into consideration to obtain the correct charge state and mean mass assignment* ***A)*** *chevron plot showing standard devation of the mean mass across all charge state peaks for different charge state assignments, represented by the charge value assigned to the first peak at 88,380 m/z. The applied assignment of peak 1 (88,380 m/z) to the 42+ charge state is circled in black; it corresponds to the lowest apparent mass increase with decreasing charge. This assignment results in a mean mass of 3764 ± 34 kDa, in agreement with the results of CDMS experiments (3.79 MDa, Figure 5A).* ***B)*** *Difference in mass between the mean mass across all charge states and the mean mass for a specific charge state peak, plotted for five different charge state assignments. A negative number means that the peak for the specific charge state is heavier than the mean mass, and a positive number means that is is lighter than the mean mass. Peak 1 corresponds to the peak at 88380 m/z. The final selected charge assignment (peak 1 = 42+, black trace) exhibits a negative correlation between charge and mass, while also having low standard deviation in mean mass standard deviation (as shown in panel A).*

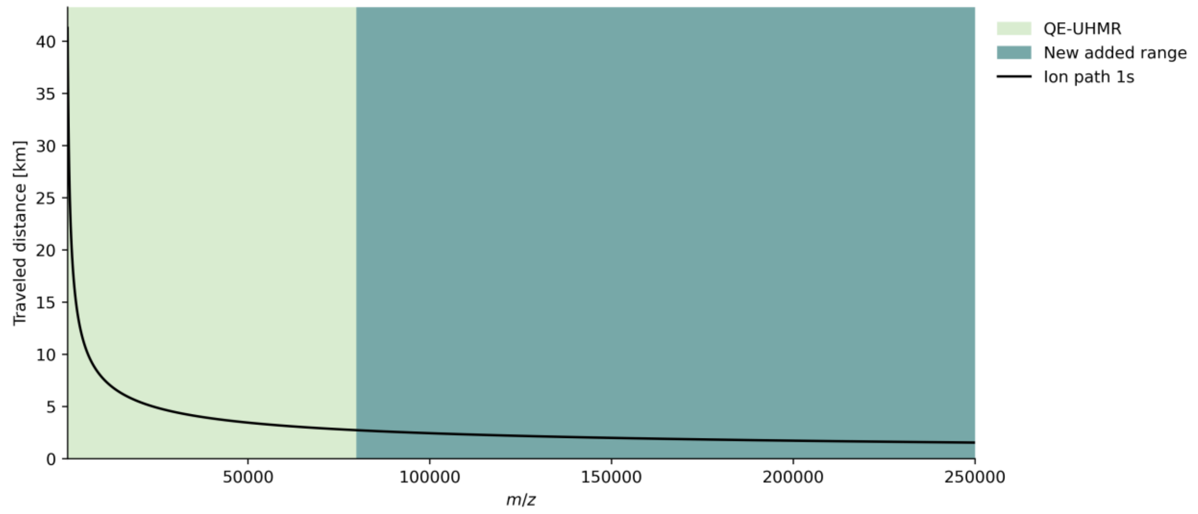

Suppl. Fig. 8. *Distance travelled vs. m/z for ions during a 1 second transient. Higher m/z ions, having lower frequencies, travel reduced distances in the Orbitrap analyser at lower velocities compared to lower m/z ions over the same transient time , resulting in fewer and less energetic collisions. The plot background is coloured to illustrate the standard m/z range of the QExactive UHMR MS and the further range newly added in this work.*

Supplementary References

1. J. Snijder, R. J. Rose, D. Veesler, J. E. Johnson, A. J. Heck, Studying 18 MDa virus assemblies with native mass spectrometry. *Angew Chem Int Ed Engl* **52**, 4020-4023 (2013).
2. F. Sigmund *et al.*, Bacterial encapsulins as orthogonal compartments for mammalian cell engineering. *Nat. Commun.* **9**, 1990 (2018).
3. D. Veesler *et al.*, Architecture of a dsDNA Viral Capsid in Complex with Its Maturation Protease. *Structure* **22**, 230-237 (2014).
4. L. F. Barnes, B. E. Draper, M. F. Jarrold, Analysis of Recombinant Adenovirus Vectors by Ion Trap Charge Detection Mass Spectrometry: Accurate Molecular Weight Measurements beyond 150 MDa. *Anal. Chem.* **94**, 1543-1551 (2022).
5. T. P. Wörner *et al.*, Adeno-associated virus capsid assembly is divergent and stochastic. *Nat. Commun.* **12**, 1642 (2021).
