# Supplementary material for: To 200,000 m/z and Beyond: Native Electron Capture Charge Reduction Mass Spectrometry Deconvolves Heterogeneous Signals in Large Biopharmaceutical Analytes
Source: ACS Cent Sci. 2024 Jul 26;10(8):1548–61. doi: 10.1021/acscentsci.4c00462 (PMC11363327; doi:10.1021/acscentsci.4c00462)
Supplement: Supplementary file 2 — oc4c00462_si_002.pdf [file oc4c00462_si_002.pdf]

oc-2024-004627.R1

Name: Peer Review Information for "To 200,000 m/z and beyond: native electron capture charge reduction mass spectrometry deconvolves heterogeneous signals in large biopharmaceutical analytes"

First Round of Reviewer Comments

Reviewer: 1

Comments to the Author

This work uses electron capture for limited charge reduction of large electrosprayed ions. The analytical benefits of this approach are discussed and illustrated using several examples. Overall, this is an interesting study, but a couple of points should be addressed:

Main Comments:

Another approach to deal with overlapping peaks and charge states is the use of deconvolution software such as UniDec or MaxEnt. Those tools are quite powerful and should not be dismissed. Not mentioning them is an oversight.

p. 9 “Simulating the effect of charge reduction on heterogeneous adeno-associated virus assemblies” and Figure 4 (also SI Figs 2 and 3): It is somewhat disappointing that the potential of the technique developed here is illustrated with simulated data, instead of showing actual experiments.

The Figure 5 data are very nice. However, to illustrate the usefulness of ECCD, it would be necessary to show how “bad” (unresolvable) these spectra are without ECCD. As it stands, readers are wondering whether perhaps charge states are already quite clear without ECCD.

p. 5: end of introduction. Readers are confused whether m/z 80,000 is a hard ceiling for the instrument used here (based on hardware and software specifications), or if it is an approximate

guess. Either way, it seems odd to state that the use of higher m/z ions (point iii) will extend the mass range of the instrument.

Minor Points and Suggestions:

“dealign” in the abstract may carry a negative connotation. Consider rewording (“separate” ?)

p. 2: “cause collisional energy transfer” change to “collisional heating”?

This work frequently uses the term “convolution” as a synonym for “overlap”. I suggest using the latter, to make this work more accessible to non-experts. Please also note that “convolution” is has a well defined mathematical meaning, that is unrelated to the use of that term in this manuscript.

https://mathworld.wolfram.com/Convolution.html

Figure 1: Caption is much too long. In-figure legends are too small. Poor choice of colors in A/B , where yellow(ish) refers to different proteins, and blue switches meaning from POPA to AmtB + n POPA. Not clear if Antibody with drug loaded in (B) refers to the same system as (C).

p. 2: “($(m/z)ion1$ = $(m/z)ion2$ = $(m/z)shared$)” Unnecessary. Delete.

p. 2: “at which Δmass complete peak overlap would occur, causing exact peak overlap” awkward wording

eq 2 is unnecessarily complicated, because z_n+1 – z_n = 1.

p. 4: “to account for the mass of one additional Dalton per reduced charge, however this additional mass” Misleading. This seems to suggest that every captured electron has a mass of 1 Da.

The foundation of SI Fig 1 is unclear. References are provided, but what equation was used?

p. 5: “low energy electrons” please specify the energy (eV) range.

p. 6: not clear why temporal descriptors are used in the text, such as “slowly” and “rapidly”

p. 7: “arbitrarily and easily” sounds odd.

Figure 2 caption. Continue to use “charge reduction”. Do not switch to “stripping”

Either use the “ECCD” acronym or “electron capture charge reduction” throughout the text, but do not switch back and forth.

Reviewer: 2

Comments to the Author

This is an excellent manuscript that describes the analytical merits of extreme reduction of charge of very large gas-phase analytes generated by electrospray ionisation mass spectrometry. The advantages of mass spectrometry measurements of charge reduced species has been outlined previously by many reports, e.g., McLuckey and his contributions towards the development of proton transfer charge reduction (PTCR), Bush and his CAPTR method, and electron-based methods, such as ETnoD, as outlined in the manuscript. By reducing charge, peaks representing multiply charged analytes can be better separated in m/z-space for their unambiguous assignment of charge (and subsequently their mass). This is especially important for large, heterogeneous analytes; a perfect example is the MDa-sized AAV8 capsids shown in this report. With charge reduction, i.e., reducing z, an analyser with increased m/z-range is required. This report also demonstrates the capability of the orbitrap analyser to measure signal beyond m/z 200,000 – the highest reported for an orbitrap.

Overall, the contents of this report represents an important analytical utility for the mass measurement of ultra-large (MDa) assemblies, for example, viral assemblies and other vehicles used for drug deliver. The ECCR method appears to be competitive to the so-called single-ion charge detection strategy.

There are very few faults that I can find in the manuscript. However, there are a few questions that could be addressed in a final revised manuscript, especially with respect to the accessibility of the overall method to other laboratories.

1. The term “slowly” is used to describe the tuning of the ExD voltages (pp. 6, 7). To what exactly is “slowly” referring? I am assuming that the “speed” to which the voltage is increased is not intended here, but rather a small step increase in voltage was used.

2. A signal intensity loss is described on page 7 as a result of ECCR. This could be further illustrated with a figure (in supplement) that shows the spectra in Fig. 2a and Fig. 3c plotted as a function of absolute intensity, rather than normalised intensity.

3. The manuscript describes the ability to increase the orbitrap m/z-range beyond m/z 100,000. What is the effective resolving power of the analyser above m/z 100,000? Is it clear that the large peak widths in the spectra shown in Figure 5 are due to desolvation problems and adduct formation, rather than lower resolving power in this m/z-range?

4. It is not clear why the authors are surprised that “virus particles are able to capture ca. 140 electrons, experience up to ca. 90% charge reduction and survive intact until detection without dissociation” (pp. 12 and 13). Given previous work by others using PTCR and other methods for charge reduction, ion dissociation does not appear to be a prevalent pathway during charge reduction.

5. Figure 5 – It might be useful to show deconvolved mass spectra alongside the CDMS spectra, e.g., using UniDec. How is m/z calibration performed at such high m/z-range?

6. Methods for charge reduction using ion-ion/neutral reactions, e.g., PTCR, and ETnoD methods exist on current orbitraps and other analysers. The manuscript clearly demonstrates that ECCR with the ExD cell is an extremely effective means for charge reduction. However, given the fact that the ExD cell is no longer commercially available to orbitrap users (because the company that produces the ExD device, eMSion, has been acquired by Agilent Technologies and they no longer offer ExD to non-Agilent customers), it is not clear that the methods described here will be widely adopted (besides by the few orbitrap users who happened to install the ExD cell before the Agilent acquisition). So, how does ECCR compare to PTCR and ETnoD for charge reduction?

Author's Response to Peer Review Comments:

Thanks for the encouraging update and please see attached feedback document

COMMENTS FROM EDITOR
Thank you for your recent submission to ACS Central Science. We have now received the reviews for your manuscript and I am pleased to inform you they were quite positive.

However, the reviewers have raised important points that require attention and must be addressed before a final decision can be made.

Please make the appropriate changes to your manuscript and submit a revised manuscript. Your manuscript may be subject to further peer review but if the revision can thoroughly address the outstanding concerns, we aim to minimize further back-and-forth correspondence between authors and referees and make editorial decisions in house, which will save time and effort for all and expedite processing of your paper.

The revision should address the reviewers' comments and include a point-by-point response. In addition to a clean copy of the revised manuscript, please also submit a tracked version of the original submission that shows the actual changes (deletions and additions) made to the manuscript. You may highlight, color font, or underline the changes.

REVIEWER 1

Recommendation:

Publish in ACS Central Science after minor revisions noted.

Comments:
This work uses electron capture for limited charge reduction of large electrosprayed ions. The analytical benefits of this approach are discussed and illustrated using several examples. Overall, this is an interesting study, but a couple of points should be addressed:

Main Comments:
Another approach to deal with overlapping peaks and charge states is the use of deconvolution software such as UniDec or MaxEnt. Those tools are quite powerful and should not be dismissed. Not mentioning them is an oversight.

> MaxEnt and UniDec are indeed powerful tools for deconvolving complex charge state distributions, even in many cases where charge states overlap. We have added a sentence to the introduction describing these methods and are grateful to the reviewer for identifying this oversight.
>
> We note however, that for these methods to be successful they require at least some partially resolved peaks which can be assigned to individual charge states. In the case of

extremely heterogeneous and stochastically assembling samples, such as adeno-associated viruses (AAVs), there are so many overlapping peaks from ions of different charges and masses contributing to the observed signal that methods such as UniDec are unable to deconvolve the true charge state distribution. In such cases more advanced techniques are required, such as charge detection mass spectrometry, which has in recent years enabled the determination of the charge state distribution of AAV samples by simultaneously measuring *m/z* and charge. Our paper introduces a novel alternative solution to this problem by extensive charge reduction to lower charge states, which separates out the previously overlapping charge states in *m/z* space.

As an illustration of the limitations of software deconvolution for AAVs, we have presented below a native mass spectrum of empty AAV8 capsids without charge reduction (left), and the result of UniDec deconvolution for this spectrum (right). UniDec is unable to correctly assign the average mass of the capsids or the mass distribution. We have included a similar native mass spectrum as a new supplementary figure 4 in the paper.

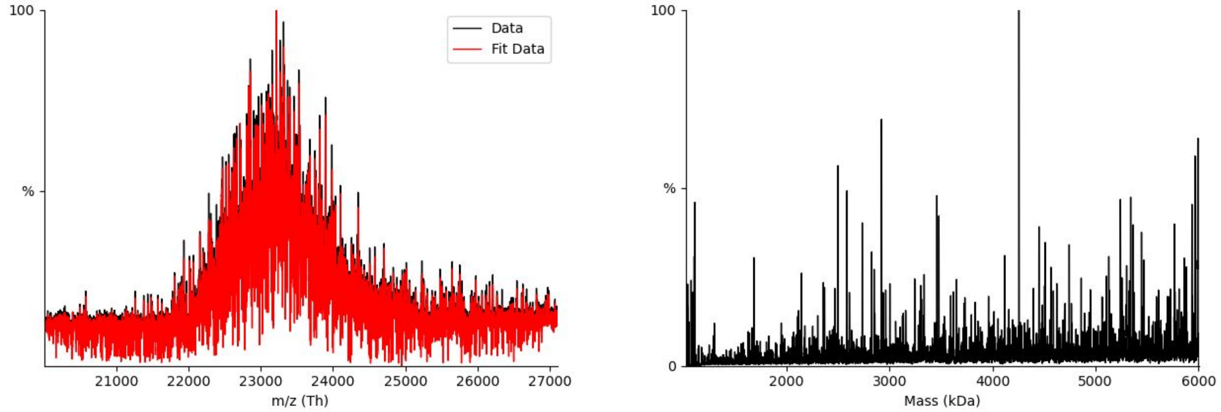

p. 9 “Simulating the effect of charge reduction on heterogeneous adeno-associated virus assemblies” and Figure 4 (also SI Figs 2 and 3): It is somewhat disappointing that the potential of the technique developed here is illustrated with simulated data, instead of showing actual experiments.

Simulations play an important role in this paper by allowing us to model and conceptualize the effects of charge reduction on protein native mass spectra. The intention of the introduction is to provide a theoretical framework, explain the concepts of charge reduction and illustrate known cases from the existing literature where charge reduction has proven useful. This is why we develop the equations in the introduction and demonstrate the underlying concept with simulated data (with simulations being based on real experimental results reported previously in the literature for smaller proteins).

Later in the results sections we show exciting new data from experiments and the effects of charge reduction on the spectra of large and heterogeneous AAV capsid (an example of

which is now included as supplementary figure, see above), as an especially challenging analyte. The simulations in Figure 4A (also SI Figs 2 and 3) are essential for providing a theoretical basis for the results for the separation overlapping charge states of AAV capsids by charge reduction; which we then demonstrate experimentally in Figure 5.

We can simulate the mass distribution of a heterogeneous mixture of the 60-mer AAV virus capsids from the three possible structural subunit proteins (VP1, VP2 and VP3) of known mass, assuming that the capsids assemble stochastically and by estimating expression ratios of the subunit proteins. There are 1089 possible unique VP1:VP2:VP3 stoichiometries, and we can model the mass probability distribution. From the mass distribution, and assuming native charging behaviour, we can simulate the probability distribution of all ions and thereby simulate a mass spectrum at a given instrument resolution. Crucially, because there are so many overlapping signals from ions of similar m/z but different mass, charge and population/probability, the simulation method allows us to model which ion signals contribute to the observed peaks in the overall mass spectrum, which is actually an interference pattern due to the overlapping signals. The simulations show that when AAVs are natively charged, under standard experimental conditions, there are up to 12 unique charge states (and multiple masses per charge state) overlapping within a single observed peak. This provides the theoretical explanation for why correct charge and mass assignments cannot be made from conventional native mass spectra of heterogeneous AAVs, because analysis methods (including software deconvolution algorithms) depend on the presence of resolved peaks with individual charge states. The simulation methodology and theoretical insight into the difficulties of analysis of AAVs been native mass spectrometry has been previously published (https://www.nature.com/articles/s41467-021-21935-5). We extend these previously published findings by showing that when the charges of the ions in the simulation are extensively reduced, the charge state populations separate into resolved peaks arising from ions of the same charge state – making it possible to assign the charge states and calculate the mean mass. **The simulations are imperative for their ability to model which charge states contribute to the observed peaks.**

Our subsequent experimental data demonstrate that we can perform the required extensive charge reduction to AAV capsids; that we can extend the upper *m/z* range of the Orbitrap UHMR mass spectrometer to measure in the 100k-250k *m/z* range required to detect AAVs that have been sufficiently charge reduced to separate the charge states; and finally that we are able to achieve the charge reduction and charge state separation of the AAVs experimentally, as predicted by the simulations; measuring mean masses which are in agreement with the results of charge-detection mass spectrometry measurements for these samples. Our paper is therefore strengthened by the combination of theory, simulations and experiments.

The Figure 5 data are very nice. However, to illustrate the usefulness of ECCD, it would be necessary to show how "bad" (unresolvable) these spectra are without ECCD. As it stands, readers are wondering whether perhaps charge states are already quite clear without ECCD.

While recent publications have already demonstrated nicely that natively charged AAV charge states are not only unresolvable (https://doi.org/10.1038/s41467-021-21935-5

https://doi.org/10.1039/D3SC03228K), but can actually produce the false appearance of charge states where there is coincidental overlap and interference of signals of different mass and charge, we agree that maybe some readers are not aware of this and decided to include our own native charging data in a new SI figure (see above). We have expanded our introduction to AAVs to further clarify these points.

p. 5: end of introduction. Readers are confused whether m/z 80,000 is a hard ceiling for the instrument used here (based on hardware and software specifications), or if it is an approximate guess. Either way, it seems odd to state that the use of higher m/z ions (point iii) will extend the mass range of the instrument.

The commercial Q Exactive UHMR product line has an upper m/z specification of 80,000 m/z, constrained by the instrument control software, in order to ensure that performance is uniform for all instruments of this line. The research-grade instrument used here without this software limit shows ion transmission and detection well above 80,000 m/z (red data in Figure 4C), demonstrating that this is not a hard ceiling. The data from Figures 2A and 4C indicate that the true hardware limit, which was previously not known, appears to be about 125,000 m/z. We further show (Figure 4C) that with instrument modifications this can be extended to at least 250,000 m/z. We have clarified this now in the text.

We agree that the sentence in the introduction regarding the extension of the m/z range sounded confusing and we have reworded it: "…use the high m/z ions thus generated to explore the upper m/z range of the UHMR, and through instrument modification extend the m/z range if possible".

Minor Points and Suggestions:

"dealign" in the abstract may carry a negative connotation. Consider rewording ("separate" ?)

It might indeed – thanks for pointing this out – and also to keep the language simple, we decided to follow the suggestion to replace this with "separate".

p. 2: "cause collisional energy transfer" change to "collisional heating"?

Since collisional energy transfer has the effect of causing collisional heating here, we followed this reviewer's suggestion to reword accordingly.

This work frequently uses the term "convolution" as a synonym for "overlap". I suggest using the latter, to make this work more accessible to non-experts. Please also note that "convolution" is has a well defined mathematical meaning, that is unrelated to the use of that term in this manuscript.

Simpler terms are always better as long as they are precise, and "overlap" would indeed be sufficiently clear here. We are therefore happy to follow the reviewer's suggestion. However we have elected to keep the term "deconvolve" (often also referred to as "deconvolute") in

the title and abstract, as unlike “convolution”, this is a term which is already commonly found in the native mass spectrometry literature.

Figure 1: Caption is much too long. In-figure legends are too small. Poor choice of colors in A/B , where yellow(ish) refers to different proteins, and blue switches meaning from POPA to AmtB + n POPA. Not clear if Antibody with drug loaded in (B) refers to the same system as (C).

We agree that the use of colours was inconsistent between the sub-panels in this figure, hindering readability. We have now modified the colour scheme to ensure clarity and consistency throughout the figure. High contrast colours are now consistently used throughout to code the two different proteins (AmtB and an antibody) and their ligands. Blue circles are used to indicate POPA lipid binding to AmtB and green circles are used to indicate drug conjugation to the antibody. We have furthermore changed the representation of the number of loaded drug molecules in panel C so that the illustration is consistent with the illustration of the number of bound lipids in panel A. The size of the in-figure legends has been increased by 10%.

The figure caption has been simplified, with the additional details moved to the main text.

p. 2: “$((m/z)ion1 = (m/z)ion2 = (m/z)shared)$” Unnecessary. Delete.

We agree that this is not necessary and have deleted it.

p. 2: “at which Δmass complete peak overlap would occur, causing exact peak overlap” awkward wording

We rephrased this to “at which Δmass complete peak overlap would occur”

eq 2 is unnecessarily complicated, because z_n+1 – z_n = 1.

In this case of adjacent charge states, this equation does indeed simplify as spotted by the reviewer. We have added a sentence to the introduction to highlight this simplification. However we have kept the non-simplified version of equation 2 as it is, because we later wish to expand this in equation 3 to the more general case of z_n+x – z_n for overlap of peaks which aren’t necessarily directly adjacent charge states, and we think it’s beneficial to keep the more complex version of equation 1 in order for the reader to see the generalization in equation 3.

p. 4: “to account for the mass of one additional Dalton per reduced charge, however this additional mass…” Misleading. This seems to suggest that every captured electron has a mass of 1 Da.

We thank the reviewer for identifying this misleading language. For clarity we have changed “the mass of one additional Dalton”, to “the mass of one additional proton” and we have added a further sentence explaining the origin of this difference: “This is because during positive mode electrospray ionisation, the protein is charged by protonation of surface-exposed amino acids. Therefore a protein ion charge reduced by electron capture to a lower charge state will carry with it additional proton mass compared to an ion of the same protein which had been ionised to the lower state without having undergone charge reduction”.

The foundation of SI Fig 1 is unclear. References are provided, but what equation was used?

We have now provided the equation $mass = 1.63 \times 10^{-3} \times m/z^{2.14}$ (where mass is in Da) and expanded the explanation in the figure legend. This empirical equation was determined by Albert Heck and colleagues for native protein ions up to 20 MDa (see https://doi.org/10.1038/s41467-018-04227-3, supplementary figure 3) and is consistent with recent observations by Martin Jarrold and colleagues of ~156 MDa virus particles natively charging with around 1100 charges (see https://doi.org/10.1021/acs.analchem.1c02439, figure 2 panel D).

p. 5: "low energy electrons" please specify the energy (eV) range.

After discussion with Joe Beckman, founder of e-MSion (now owned by Agilent) which developed the ExD cell, we believe that the electron energy is likely below 3 eV and this has now been added to the text.

p. 6: not clear why temporal descriptors are used in the text, such as "slowly" and "rapidly"

We agree that the temporal descriptions with regard to tuning of the ExD cell voltages were somewhat ambiguous, and describe now more precisely that small step increases in voltage were used. With regard to the experiments comparing the upper *m/z* range before and after instrument modification, we have kept the description "rapidly" in the sentence "the Orbitrap configuration was then rapidly switched to the modified configuration with reduced voltage ramp rate and electrospray was restarted from the same needle, in the same position, with all other instrument settings the same and acquired again for four minutes". This is because we purposefully carried out the instrument modification quickly (~2 minutes), which required shutting down the instrument and modifying the hardware, in order to immediately restart the electrospray and a new acquisition with the same capillary, sample, and capillary positioning to ensure comparable conditions between the spectra acquired before and after the modification.

p. 7: "arbitrarily and easily" sounds odd.

We reworded this to "arbitrarily".

Figure 2 caption. Continue to use "charge reduction". Do not switch to "stripping"

Agreed – we replaced "stripping" with "charge reduction".

Either use the "ECCD" acronym or "electron capture charge reduction" throughout the text, but do not switch back and forth.

We think that it is ok to use these terms interchangeably throughout the text after the acronym has been defined, depending on the flow of the sentence.

REVIEWER 2

Recommendation: Publish in ACS Central Science after minor revisions noted.

Comments:

This is an excellent manuscript that describes the analytical merits of extreme reduction of charge of very large gas-phase analytes generated by electrospray ionisation mass spectrometry. The advantages of mass spectrometry measurements of charge reduced species has been outlined previously by many reports, e.g., McLuckey and his contributions towards the development of proton transfer charge reduction (PTCR), Bush and his CAPTR method, and electron-based methods, such as ETnoD, as outlined in the manuscript. By reducing charge, peaks representing multiply charged analytes can be better separated in m/z-space for their unambiguous assignment of charge (and subsequently their mass). This is especially important for large, heterogeneous analytes; a perfect example is the MDa-sized AAV8 capsids shown in this report. With charge reduction, i.e., reducing z, an analyser with increased m/z-range is required. This report also demonstrates the capability of the orbitrap analyser to measure signal beyond m/z 200,000 – the highest reported for an orbitrap.

Overall, the contents of this report represents an important analytical utility for the mass measurement of ultra-large (MDa) assemblies, for example, viral assemblies and other vehicles used for drug deliver. The ECCR method appears to be competitive to the so-called single-ion charge detection strategy.

There are very few faults that I can find in the manuscript. However, there are a few questions that could be addressed in a final revised manuscript, especially with respect to the accessibility of the overall method to other laboratories.

1. The term "slowly" is used to describe the tuning of the ExD voltages (pp. 6, 7). To what exactly is "slowly" referring? I am assuming that the "speed" to which the voltage is increased is not intended here, but rather a small step increase in voltage was used.

   We agree that the wording was somewhat ambiguous, and describe now more precisely that small step increases in voltage were used.

2. A signal intensity loss is described on page 7 as a result of ECCR. This could be further illustrated with a figure (in supplement) that shows the spectra in Fig. 2a and Fig. 3c plotted as a function of absolute intensity, rather than normalised intensity.

   We agree that this would be good to show here and have included a new supplementary figure illustrating the signal intensity loss.

3. The manuscript describes the ability to increase the orbitrap m/z-range beyond m/z 100,000. What is the effective resolving power of the analyser above m/z 100,000? Is it clear that the large peak widths in the spectra shown in Figure 5 are due to desolvation problems and adduct formation, rather than lower resolving power in this m/z-range?

While the theoretical resolving power could be calculated from the transient length for this m/z range, in reality the resolution will be limited by the extent of desolvation for these ions. This is even more the case for extremely charge reduced ions in this paper, as they experience less acceleration in the HCD cell due to their reduced charge. We discuss this in further detail in our response to point 5.

4. It is not clear why the authors are surprised that “virus particles are able to capture ca. 140 electrons, experience up to ca. 90% charge reduction and survive intact until detection without dissociation” (pp. 12 and 13). Given previous work by others using PTCR and other methods for charge reduction, ion dissociation does not appear to be a prevalent pathway during charge reduction.

We are indeed not too surprised as we saw a comparable extent of charge reduction in previous work on ADH and other protein complexes with “crETD” (charge reduction ETD, ETnoD) on the Synapt instrument (https://pubs.acs.org/doi/10.1007/s13361-015-1124-z), and larger particles present larger cross sections for electron capture – but we, like probably many readers, find it nevertheless remarkable that increasingly charge-deprived particles continue to react effectively with more electrons within the time frame of the ion/electron interaction until 90% of initial charge is neutralized. We want to be more precise though with our statement and modified the text to make clear that this observation – while remarkable – is nevertheless broadly in line with expectations.

5. Figure 5 – It might be useful to show deconvolved mass spectra alongside the CDMS spectra, e.g., using UniDec. How is m/z calibration performed at such high m/z-range?

We have added a new supplementary figure illustrating the charge assignment/deconvolution analysis for these spectra. We have used the “chevron” method, used previously for charge state assignments of large virus assemblies (https://pubs.acs.org/doi/10.1021/ja993740k), which aims to find the assignment which minimizes the standard deviation in mass across all the peaks in the series. Crucially, we also add the constraint of differential desolvation, which takes into account the fact that the lower charge state ions will exhibit slightly higher apparent mass than more highly charged ions, because they experience less acceleration and therefore less desolvation in the HCD cell. The normally negligible effect of differential desolvation is made more prominent in this case because of the large solvent mass associated with the megadalton virus particles and the extensive charge reduction.

The question of mass accuracy and calibration at these high m/z ranges is interesting. In this work the instrument was calibrated for mass using the standard UHMR calibration procedure with monoisotopic cesium iodide clusters. Because all ions move around at approximately the same average radius inside the Orbitrap, we can expect that calibration for moderate *m/z* CsI clusters would also hold for the high *m/z* range within ppm levels. It is however challenging to determine the mass accuracy at high *m/z* for the analytes studied in this manuscript, as not only will each individual ion carry a different additional mass of residual solvent, the AAV capsid samples are furthermore highly heterogeneous in mass due to the 1089 possible subunit compositions. Therefore we do not have an accurate

reference mass for each ion from which we could calculate the mass accuracy. Furthermore, it is known that the empirical widths of native mass spectral peaks exceed the theoretical widths defined by the instrument resolution, due to the incomplete desolvation of these ions, such that the mass accuracy only approaches that of the nominal mass (https://doi.org/10.1021/acs.chemrev.1c00212). In this work incomplete desolvation is an even greater issue, due to the extensive charge reduction (less desolvation in the HCD cell) and high mass (i.e., more residual solvent) of the particles. Considering these facts, we can expect that any deviation from the mass calibration at high m/z will be an insubstantial factor compared to the peak broadening consequent from poorer (and differential) desolvation of the highly charged reduced ions. Nevertheless, Figure 5 shows reasonable agreement in the average masses compared between the charge reduction and charge detection mass spectrometry measurements.

6. Methods for charge reduction using ion-ion/neutral reactions, e.g., PTCR, and ETnoD methods exist on current orbitraps and other analysers. The manuscript clearly demonstrates that ECCR with the ExD cell is an extremely effective means for charge reduction. However, given the fact that the ExD cell is no longer commercially available to orbitrap users (because the company that produces the ExD device, eMSion, has been acquired by Agilent Technologies and they no longer offer ExD to non-Agilent customers), it is not clear that the methods described here will be widely adopted (besides by the few orbitrap users who happened to install the ExD cell before the Agilent acquisition). So, how does ECCR compare to PTCR and ETnoD for charge reduction?

Since the completion of this experimental work the specific ExD device used in this manuscript is indeed now, at least for the time being, commercially available only for Agilent instruments. However the findings described here are of much wider importance to the field and are independent of the specific device, method of charge reduction, or type of mass spectrometer used, and will be of interest to a broad audience.

We furthermore expect that the utility and general methodology of native charge reduction described in our paper will also be applicable for charge reduction achieved by ETnoD and PTCR; indeed this work follows on from of our previous study demonstrating on a Synapt mass spectrometer that native proteins and complexes could be charge reduced to 100,000 *m/z* with “crETD” or ETnoD (https://pubs.acs.org/doi/10.1007/s13361-015-1124-z). A detailed comparison of the effectiveness of ECCR, ETnoD and PTCR would be an interesting further study but is beyond the scope of the current work.

Furthermore, independent of the subject of charge reduction, our new high *m/z* capabilities of the UHMR are an exciting development for orbitrap mass spectrometry in general, as it shows it is now possible to study even heavier assemblies which natively charge well above 100,000 m/z, such as large viruses (adenovirus) and virus-like particles which are becoming increasingly important biopharmaceutical analytes.

We have added text to our discussion highlighting the broad applicability of our findings.

FORMATTING NEEDS FROM JOURNAL

SI PARAGRAPH: If the manuscript is accompanied by any supporting information for publication, a brief description of the supplementary material is required in the manuscript. The appropriate format is: Supporting Information. Brief statement in non-sentence format listing the contents of the material supplied as Supporting Information.

We added this short statement about the supplementary data.

TOC MISSING: Provide a TOC image per journal guidelines (3.25 in. × 1.75 in. (8.25 cm × 4.45 cm) ; on the last page of the Manuscript) with the heading “TOC Graphic” above the graphic. Make sure to designate the file as “Graphic for Manuscript.”

TOC graphic added and adheres to journal size guidelines.

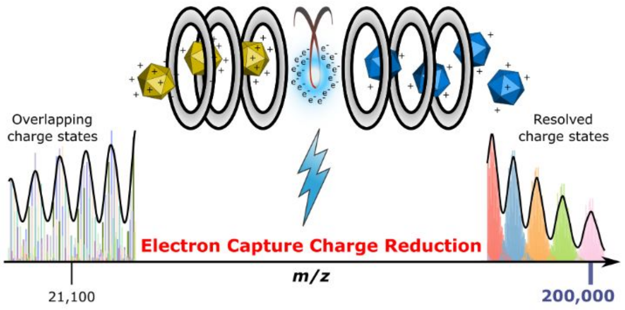

SYNOPSIS MISSING: The synopsis should be no more than 200 characters (including spaces) and should reasonably correlate with the TOC graphic. The synopsis is intended to explain the importance of the article to a broader readership across the sciences. Please place your synopsis in the manuscript file after the TOC graphic, and label it as “Synopsis.”

Synopsis added.

CAPTION PERMISSIONS: If any of your graphics have been previously published this should be stated in the caption and copyright permissions should be obtained from any non ACS publications. Upload copies of the permissions as Other Files for Editors Only with your revision.

Please note: If you have permitted use as the author of the previously published graphics, you do not need to supply written copyright permission.

Credit lines should read as follows: “Reproduced [or Adapted, Reprinted, etc.] with permission from Ref. [number], Copyright [year] [journal name].”

SI FILE: Please provide as a separate file, designated SI for Publication. Do not include your SI in the MS file.

SI now submitted as a separate file.
